# Mechanisms of Cell Cycle Control Revealed by a Systematic and Quantitative Overexpression Screen in *S. cerevisiae*

**DOI:** 10.1371/journal.pgen.1000120

**Published:** 2008-07-11

**Authors:** Wei Niu, Zhihua Li, Wenjing Zhan, Vishwanath R. Iyer, Edward M. Marcotte

**Affiliations:** 1 Center for Systems and Synthetic Biology, University of Texas, Austin, Texas, United States of America; 2 Institute for Cellular and Molecular Biology, University of Texas, Austin, Texas, United States of America; 3 Section of Molecular Genetics and Microbiology, University of Texas, Austin, Texas, United States of America; 4 Department of Chemistry and Biochemistry, University of Texas, Austin, Texas, United States of America; Yale University, United States of America

## Abstract

Regulation of cell cycle progression is fundamental to cell health and reproduction, and failures in this process are associated with many human diseases. Much of our knowledge of cell cycle regulators derives from loss-of-function studies. To reveal new cell cycle regulatory genes that are difficult to identify in loss-of-function studies, we performed a near-genome-wide flow cytometry assay of yeast gene overexpression-induced cell cycle delay phenotypes. We identified 108 genes whose overexpression significantly delayed the progression of the yeast cell cycle at a specific stage. Many of the genes are newly implicated in cell cycle progression, for example *SKO1*, *RFA1*, and *YPR015C*. The overexpression of *RFA1* or *YPR015C* delayed the cell cycle at G2/M phases by disrupting spindle attachment to chromosomes and activating the DNA damage checkpoint, respectively. In contrast, overexpression of the transcription factor *SKO1* arrests cells at G1 phase by activating the pheromone response pathway, revealing new cross-talk between osmotic sensing and mating. More generally, 92%–94% of the genes exhibit distinct phenotypes when overexpressed as compared to their corresponding deletion mutants, supporting the notion that many genes may gain functions upon overexpression. This work thus implicates new genes in cell cycle progression, complements previous screens, and lays the foundation for future experiments to define more precisely roles for these genes in cell cycle progression.

## Author Summary

All cells require proper cell cycle regulation; failure leads to numerous human diseases. Cell cycle mechanisms are broadly conserved across eukaryotes, with many key regulatory genes known. Nonetheless, our knowledge of regulators is incomplete. Many classic studies have analyzed yeast loss-of-function mutants to identify cell cycle genes. Studies have also implicated genes based upon their overexpression phenotypes, but the effects of gene overexpression on the cell cycle have not been quantified for all yeast genes. We individually quantified the effect of overexpression on cell cycle progression for nearly all (91%) of yeast genes, and we report the 108 genes causing the most significant and reproducible cell cycle defects, most of which have not been previously observed. We characterize three genes in more detail, implicating one in chromosomal segregation and mitotic spindle formation. A second affects mitotic stability and the DNA damage checkpoint. Curiously, overexpression of a third gene, *SKO1*, arrests the cell cycle by activating the pheromone response pathway, with cells mistakenly behaving as if mating pheromone is present. These results establish a basis for future experiments elucidating precise cell cycle roles for these genes. Similar assays in human cells could help further clarify the many connections between cell cycle control and cancers.

## Introduction

The budding yeast *Saccharomyces cerevisiae* undergoes a cell cycle similar to other eukaryotic organisms except for the lack of nuclear envelope dissolution during mitosis and the production of daughter cells *via* budding, and thus budding yeast has become a model system for studying eukaryotic cell cycle progression [1] due to its rapid division, the availability of genetic tools, and homology to higher eukaryotic cell cycle processes. Numerous genes and proteins are involved in directing cells through the 4 major cell cycle phases, the growth gap phase G1, the DNA synthesis (S) phase, a second growth gap phase G2, and the mitotic (M) cell division phase [2],[3]. Extensive effort has been made to decipher the mechanisms of cell cycle control. However, given the extreme complexity of the cell cycle, with ∼300–800 genes regulated in a cell cycle-dependent manner [4]–[6], the complete set of cell cycle regulators, effectors, and helper proteins has yet to be determined.

Classically, conditional temperature-sensitive mutants have been very effective for studying yeast cell cycle division. Hartwell and colleagues identified more than 50 cell division cycle (CDC) genes required at specific stages in cell cycle division, by identifying conditional temperature-sensitive mutants with specific arrest points [7]–[10]. Gene dosage has been another powerful approach to study gene function. Either increasing (overexpression) or decreasing gene dosage (gene deletion or gene knockdown) can influence the activity of genes and lead to detectable phenotypes. Most large-scale cell cycle screens have focused on studying cell cycle progression by employing loss-of-function approaches such as gene deletion, RNAi, and promoter shutoff [11]–[13] and have successfully identified many cell cycle genes. However, loss-of-function mutations can often be masked, such as in the cases of genes acting as negative regulators or genes compensated for by redundant functions [14]–[16]. In contrast, overexpression of a gene product can potentially overcome such effects and often leads to a more detectable effect on cellular function [16]. Overexpression also offers the opportunity to identify and study gain-of-function mutations.

In order to identify additional cell cycle genes, especially those difficult to identify in loss-of-function studies, large-scale screens focusing on the effects of overexpression-induced gain-of-function of genes in cell-cycle progression are needed. Stevenson *et al.* performed the first such large-scale overexpression screen for cell cycle genes by expressing a moderated GAL promoter-driven cDNA library and sheared genomic DNA pool in ARS-CEN vectors [17]. Although 113 genes, including those causing only slight effects on the cell cycle, were identified from this screen, this screen was unsaturated due to the coverage of the cDNA library and incomplete gene annotation. Therefore, completion of the *S. cerevisiae* genome sequence and the systematic cloning of all genes into overexpression vectors now allow a more comprehensive analysis of the set of genes.

Analysis of overexpression phenotypes using cell sorting to assay the distribution of cells in different cell cycle stages has the advantage of being more quantitative and discerning than simple growth screens. However, flow cytometry has not been carried out comprehensively to cover all genes in the genome. In the present work, we performed a near-saturating screen for yeast genes having overexpression-induced defects in cell cycle progression, taking advantage of the availability of a yeast open reading frame (ORF) clone collection covering 91% of the yeast complete ORF set, including dubious ORFs [14]. After measuring the fraction of cells in different phases of the cell cycle *via* high-throughput flow cytometry for each of 5,556 individual ORFs and performing secondary validation assays, we identified 108 genes whose overexpression leads to significant changes in the timing of passage through the G1 or G2/M stages of the cell cycle. 82 of these genes are newly implicated in the cell cycle, with the majority likely to affect cell cycle progression *via* gain-of-function mechanisms.

## Materials and Methods

### Yeast Strains

The yeast ORF collection was obtained from Open Biosystems, in which each ORF was cloned into a 2μ plasmid under control of the GAL1 promoter in order to provide highly elevated expression when supplemented with galactose [14]. Control strains were constructed by transforming the empty precursor vector BG1766 to the ORF host strain Y258 (*MATa pep4-3, his4-580, ura3-53, leu2-3,112*) and plating on synthetic complete medium lacking uracil.

The plasmid P_GAL1_-*SKO1* was also transformed into *ste2*Δ, *ste4*Δ, *ste5*Δ, *ste20*Δ, *ste11*Δ, *fus3*Δ, *far1*Δ, *fus1*Δ, *kar4*Δ, *sst2*Δ, *dig2*Δ deletion strains [18] (ResGen/Invitrogen) and a Fus1-GFP strain [19] ( Invitrogen), as well as their parent strain BY4741 (MATa *his3*Δ *leu2*Δ *met15*Δ *ura3* and then plated on synthetic complete medium lacking uracil.

### Induction of Expression

Yeast ORF strains were induced in parallel with the corresponding empty vector (BG1766) control strain. Cells were initially grown in 96-well plates (Corning 3595) with 170 µl SD-URA medium for 1–2 days at 30°C, and then 5 µl cells were inoculated into fresh 96-well plates with 170 µl SC-URA, 2% raffinose medium. After 12 hours growth in raffinose medium, cells were re-inoculated to fresh plates with 100 µl SC-URA, 2% raffinose medium at a final O.D._600nm_ of 0.15 and grown for 1 hour. 70 µl SC-URA medium with 5% galactose (final concentration 2%) was added, and cells were grown for 8–10 hours at 30°C.

### High-Throughput Flow Cytometry

Flow cytometry analyses were performed as in [20]. Briefly, ∼2×10^6^ cells were harvested and fixed in 200 µl 70% ethanol, treated with 1mg/ml RNAse A (Sigma) for 4 hours at 37°C, then incubated with 1mg/ml Proteinase K (Sigma) for 1 hour at 50°C. ∼8×10^5^ cells were then resuspended in 200 µl 50 mM sodium citrate with Sytox green (Invitrogen) at a final concentration of 1.5 µM, performing the above liquid transfers using a Biomek FX robot (Beckman Coulter). Samples were analyzed by flow cytometry, using a Becton Dickinson FACSCalibur with BD HTS auto sampler, controlled by Plate Manager and Cellquest pro software (BD Biosciences). Well-to-well contamination was minimized by flushing with ddH_2_O between each pair of samples. In order to maximize measured events while minimizing data collection time for 5,556 strains, we collected the shorter of either 20,000 events/strain or 30-seconds acquisition time/strain. Thus, for the extremely slow growing strains, the number of events collected in 30 seconds may drop below 20,000 events.

### Analysis of Flow Cytometry Profiles

**Figure 1:**
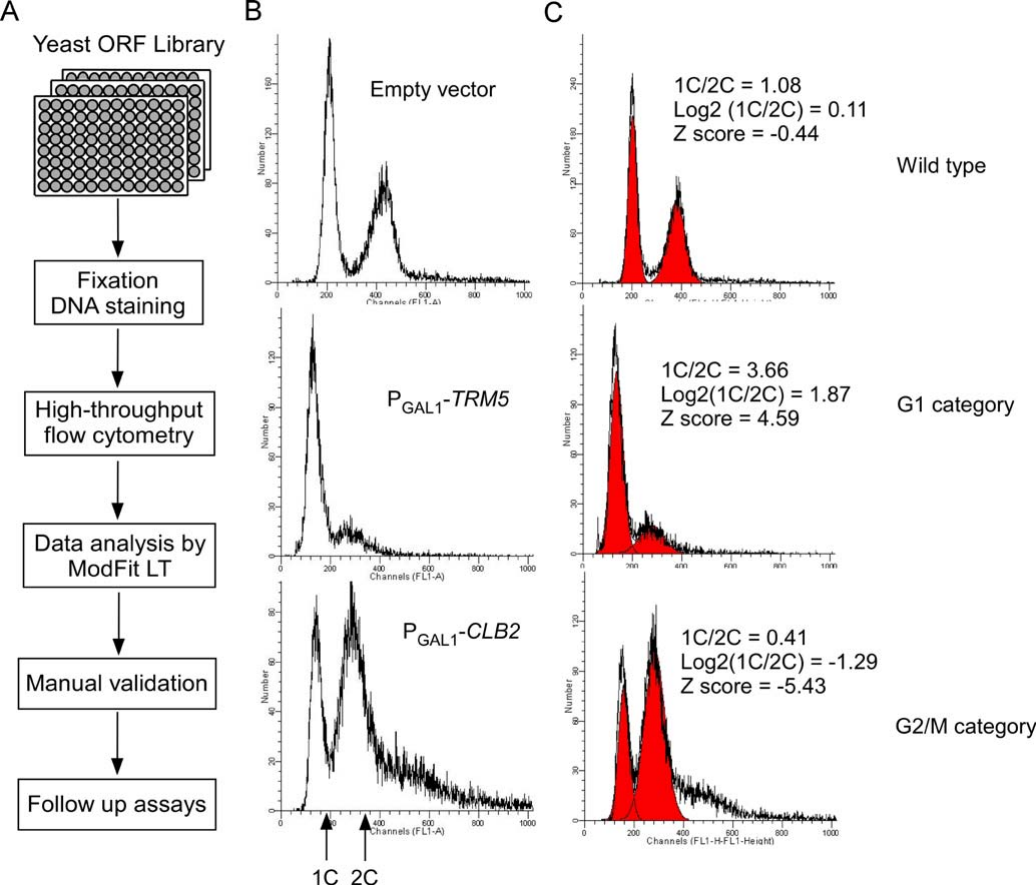
Overview of the cell cycle screen. (A) Flowchart summarizing the large-scale screen. 5,556 yeast ORF overexpression strains and 140 replicates of the empty vector (BG1766) control strain (Y258) were induced in 96-well plates with SC-URA, 2% galactose medium, and analyzed *via* high-throughput flow cytometry. All flow cytometry histograms were analyzed by ModFit LT software to calculate the proportions of cells with one copy (1C) or two copies (2C) of their chromosomal DNA. Cell cycle defects were diagnosed from skews in the proportions of 1C to 2C cells. The ORF strains that showed cell cycle defects in the initial large-scale screen were validated twice manually by flow cytometry. (B) Flow cytometry histograms of control strains and representative ORF strains are shown. The x-axis indicates fluorescence intensity, corresponding to DNA content per cell; the numbers of cells with each given intensity are plotted along the y-axis. (C) Each DNA histogram was fitted with two Gaussian distributions, shown in red, and the percentages of cells in G1 and G2/M phases were calculated as the areas under the 1C and 2C peaks, respectively.

**Figure 2:**
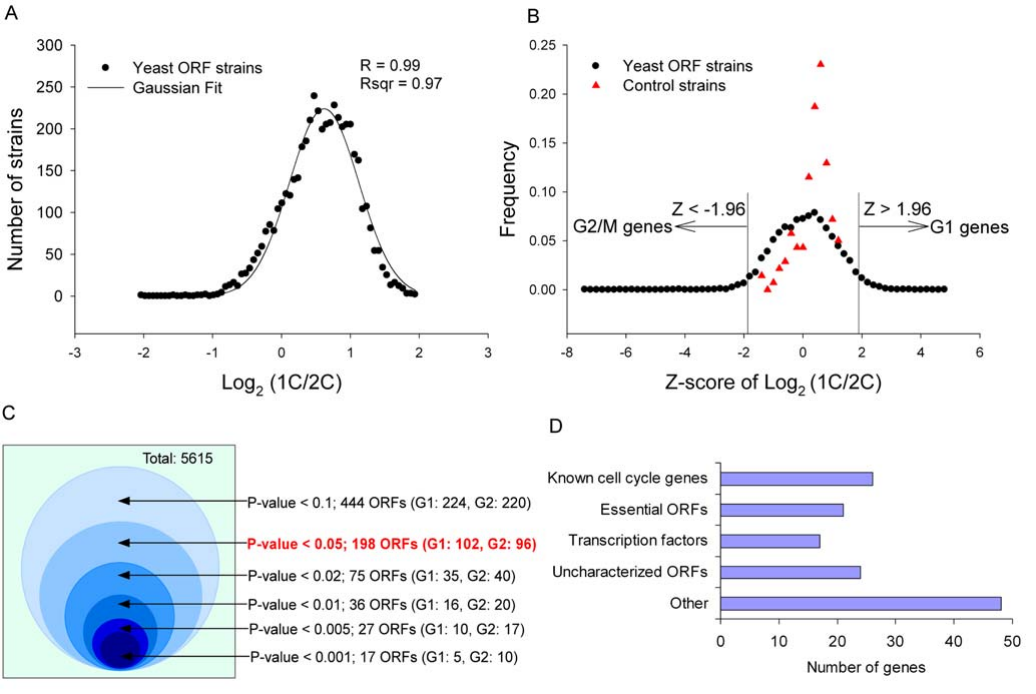
Summary of assay results. (A) The Log2 (1C/2C) ratios of the 5,334 yeast ORF strains with event numbers >5,000 (filled circles; each represents a bin of width 0.06) were approximately normally distributed and fit by a Gaussian distribution (solid line; R^2^∼0.97). Each strain was assigned a Z-score based upon its Log2 (1C/2C) ratio in order to identify the ORF strains with significantly different proportions of cells in the G1 and G2/M cell cycle phases. (B) A comparison of the resulting distribution of Z-scores for the ORF strains (filled black circles; each represents a bin of width 0.2) relative to Z-scores calculated for the replicate empty vector control strains (filled red triangles) shows that the control strains have a considerably narrower distribution than the ORF strains, with no control strain |Z| scoring higher than 1.96. (C) The numbers of ORF strains showing significantly divergent rations of 1C to 2C cells in the initial screen as a function of different confidence levels. The P-value in the paper is highlighted in bold red text; there were 198 ORF strains identified at this confidence level (*p*<0.05). Of the 198 genes, 108 were validated at least twice manually; 90 were eliminated for poor reproducibility. (D) The functional classification of the 108 genes reproducibly inducing cell cycle delays upon overexpression.

Analysis of DNA profiles was automated using ModFit 3.0 software (Verify Software house, Inc), fitting the histograms of 1C and 2C cells with Gaussian distributions (Figure 1C) and calculating the goodness-of-fit *via* the Reduced Chi Square (RCS) method. For quality control, DNA profiles with RCS>5 and event number<5000 were discarded. Empirically, we observed the resolution of the S phase cell distribution to not be of sufficiently high quality to merit systematic analysis; we thus focused instead on the well-resolved G1 and G2/M phase cells. The percentage of cells under each DNA peak (1C peak or 2C peak) was calculated by dividing the number of events under each peak by the total number of events under all peaks, and the ratio (1C/2C) of the percentage of cells under the 1C peak to that under the 2C peak was calculated for each strain. The base 2 logarithm of the 1C/2C ratio was calculated for each strain; the distribution of Log2 (1C/2C) values (abbreviated LR below) was fit well by a Gaussian distribution (R^2^ = 0.97) (Figure 2A), allowing each ORF strain *i* to be assigned a Z-score, calculated as (LR*_i_*−<LR>)/σ_LR_.

Additionally, we manually categorized strains as diploid and 3C: 208 strains appeared diploid (e.g., had 2C and 4C peaks, rather than 1C and 2C) based upon the flow cytometry data and 56 strains showed notable 3C peaks and were assigned into the 3C category. Follow-up validation of these trends showed that the DNA content of these strains did not change upon galactose induction, suggesting these to be artifacts of these strains rather than an inducible effect of gene overexpression, and thus these strains were not studied further. These strains are listed in Table S6.

### Nuclear Staining and Bud Size Measurements

108 ORF strains showing reproducible cell cycle arrest were grown and induced as described above. After induction, cells were fixed in 70% ethanol and treated with 1mg/ml RNAse A (Sigma), and then stained with 1 µM Sytox green (Invitrogen). Cells were examined *via* phase contrast microscopy and fluorescence microscopy using a Nikon Eclipse 800 fluorescence microscope. From differential interference contrast (DIC) images, we used ImageJ software (National Institute of Mental Health) to measure the length of the bud and mother cell for an average of 100 cells for each of the 108 strains. Bud size was assigned by dividing the bud length by the length of mother cell. Cells with a ratio of 0 were classified as ‘no bud’; cells were categorized into ‘small bud’ when the ratio was between 0 and 0.4, and ‘large bud’ when the ratio was higher than 0.4 [7]. We further examined the large-budded cells and counted three types of nuclear morphology: an undivided nucleus in one cell body (class I), an undivided nucleus in the bud neck (class II), and divided nuclei in two cell bodies (class III) [21]–[23]. An average of 50 cells was counted for each of 87 G2/M strains.

### Growth Assays

The 77 of 82 genes not previously implicated in cell cycle defects (and 3 positive controls, *TUB2*, *PAC2*, and *CST9*) were assayed for growth defects in three conditions: SC-URA, 2% galactose; SC-URA, 2% galactose plus 15 µg/ml nocodazole, and SC-URA, 2% galactose plus 50 µM hydroxyurea [15],[24]. 4 dubious ORFs (*YLL066W-B*, Y*BR131C-A*, *YLR123C*, *YJL077W-A*) were not included in the growth assays, as well as one gene (*PPZ1*) in the G2/M category. Cells were grown overnight in SD-URA medium, and then washed with SC-URA, 2% raffinose medium and grown in SC-URA, 2% raffinose medium for one hour at 30°C before being spotted onto agar plates. Six 10-fold serial dilutions were made for each strain, with the O.D._600nm_ of the first series at 0.2. 10 µl of each series was spotted onto SC-URA, 2% galactose plates and SC-URA 2% galactose plates containing the appropriate drugs, and grown at 30°C. Plates were photographed after 2–3 days growth in SC-URA, 2% galactose plates, or 5–8 days in the plates supplemented with drugs.

### Mitotic Instability Assay

The plasmids P_GAL1_-*YPR015C* and pRS412::ADE2 [cir+] were transformed into the strain Cry1 (MATa *ade2-1, ura3-1, leu2-3, 112, trp1, his3-11*), plating transformants on synthetic complete medium lacking uracil and adenine. A single colony was picked and diluted in ddH_2_O. ∼10^4^ cells were inoculated into SC-URA, 2% galactose medium and grown for 10 generations at 30°C, before plating ∼200 cells on a YPD plate. After growing 2–3 days at 30°C, plates were shifted to 4°C to maximize the color changes. Red and white colonies were counted, where red colonies have lost the centromere-containing plasmid and white colonies have retained it.

### Microarray Expression Profiling

The *SKO1* overexpression strain was induced in parallel with the corresponding empty vector (BG1766) control strain with 2% galactose in selective medium for 8 hours, as described above. Total RNA isolation and processing, microarray hybridization, and data analysis were performed as described previously [25], hybridizing RNA isolated from the *SKO1* ORF strain against RNA from the empty vector control strain. For each strain, two biological replicates were analyzed, each by two technical (array) replicates. Differentially expressed genes were selected as having a minimum expression ratio (corresponding to the absolute value of Log(base2) of R/G normalized ratio (Median)) > = 1.5 for at least 2 arrays. The significance of differential expression was calculated using the error model of Hughes *et al*. [26].

### Immunofluorescence Microscopy

Yeast cells were induced 8 hours, then fixed in growth medium with 1/10 volume 37% formaldehyde for 1 hour at 30°C. Fixed cell cultures were spheroplasted with 0.025 mg/ml zymolyase 20T (Seikagaku corporation) for 1 hour at 30°C. Cells were then spotted onto poly-L-lysine coated microscope slides. Cells on the slide were permeablized in −20°C methanol for 6 minutes, followed by −20°C acetone for 30 seconds. Cells were blocked with 3%BSA in PBS for 30 minutes at 30°C in a humid chamber, followed by incubation with 4 µg/ml mouse anti alpha-tubulin monoclonal primary antibodies (Invitrogen) for 1 hour and 4 µg/ml Texas Red conjugated goat anti-mouse secondary antibody (Invitrogen) for 2 hours at 30°C. After washing three times with PBS, cells were mounted with 60 µl VECTASHIELD hard set mounting medium with 1.5 µg/ml DAPI (Vector Laboratories, Inc), and imaged at 100x magnification with a Nikon Eclipse 800 microscope.

## Results/Discussion

### High-Throughput Flow Cytometry and Automated Analysis of DNA Profiles

To analyze the effect of overexpression of yeast genes on cell cycle progression, we applied high-throughput flow cytometry to screen 5,556 strains of a yeast ORF collection [14] for genes that induce delay or arrest at particular cell cycle stages when overexpressed. Figure 1 outlines the overall approach. Excess accumulation of cells with either one copy (1C) or two copies (2C) of DNA content indicates a defect in progression through a particular cell cycle stage (G1 or G2/M, respectively). Thus, in order to search for such defects induced by overexpression of a particular yeast gene, we analyzed asynchronous cell cultures and determined the distributions of DNA content, assaying if cells from each given ORF overexpression strain exhibited a skewed distribution relative to control cells. In all, ∼5,700 DNA histograms were acquired and quantitatively analyzed, measuring the ratio of 1C/2C cells for each strain, *i.e*., the ratio of cells in the G1 phase to cells in the G2/M phase. We observed the Log2 (1C/2C) ratios of the 5,556 ORF strains and of 139 replicate analyses of control strains to be approximately normally distributed and well-fit by a Gaussian distribution (R^2^∼0.97) (Figure 2A). Therefore, for each strain, we calculated a Z-score for its distribution of DNA content across cells and could thus identify the ORF strains with significantly higher accumulations of cells in the G1 or G2/M growth phases. Based on this Z-score, 2 categories were assigned: ORF strains with Log2 (1C/2C) ratios in the left tail of the Gaussian distribution were considered to have significant G2/M delays, in which cells accumulated with two copies of DNA. Similarly, ORF strains with Log2 (1C/2C) ratios in the right tail of the distribution showed significantly higher proportions of cells with one copy of DNA, and were considered to exhibit G1 delays. Examples are shown in Figure 1C. We could assign genes to the G1 and G2/M categories using different confidence levels (Figure 2B). At the 95% confidence level, 198 genes were identified whose overexpression caused cell cycle detects; only 3 of 139 control strains exceeded this threshold. As the large-scale screen was based upon only a single culture per ORF strain, we further selected those strains with reproducible defects. Of the 198 strains, 108 were validated at least twice by manual flow cytometry analysis (DNA histograms are shown in Figure S1). Additionally, we tested that all 108 genes identified showed cell cycle delay phenotypes only upon induction in galactose, and that the phenotype for each hit therefore derived specifically from the GAL-promoter-driven gene. Of the 108 genes, 21 caused a significant accumulation of cells in the G1 phase, 87 genes in the G2/M phase. These genes are listed in full in Table S1.

### Independent Validation by Bud Size Measurements

**Figure 3:**
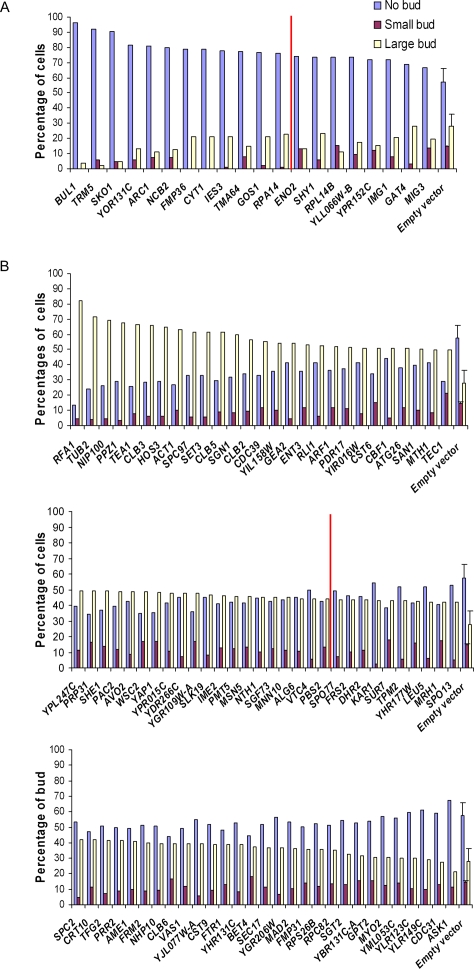
Independent support for cell cycle delays from histograms of the percentages of cells with no bud, small bud or large bud. (A) shows bud size measurements of strains in the G1 category. Strains are sorted by their percentages of cells without buds. All strains show higher proportions of cells than control strains; 13 strains are more than two standard deviations higher (indicated by red line) than the empty vector control strains (plotted+/−1 s.d.). (B) shows bud size measurements of strains in the G2/M category. Strains are sorted by percentages of cells with large buds. 85 strains showed higher percentages of large-budded cells than empty vector control strains; 47 of these were more than two standard deviations above control strains (indicated by red line). In all plots, ORF gene names are indicated in x-axis, percentages of cells on the y-axis.

**Figure 4:**
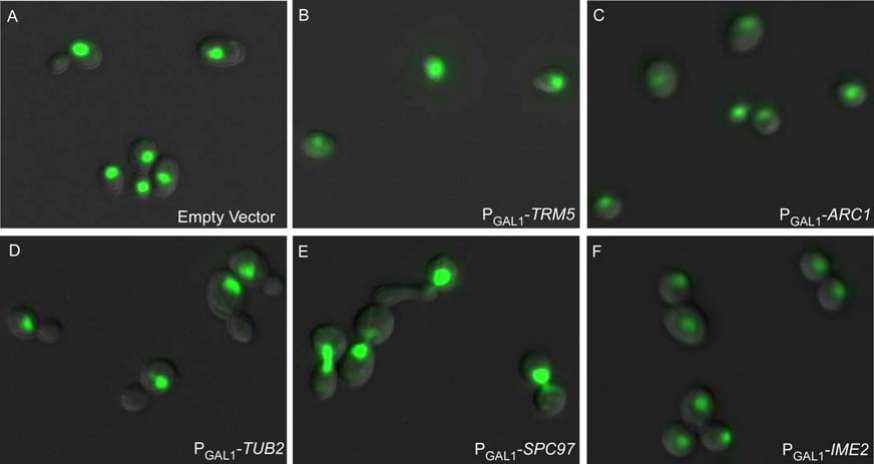
Representative cell images from ORF strains showing G1 or G2/M cell cycle delays. Cell nuclei were stained with Sytox green, and cells visualized through FITC and DIC filters; overlaid images are shown. (A) Empty vector control strain. Overexpression of (B) *TRM5* or (C) *ARC1* causes G1 cell cycle delays, marked by an accumulation of unbudded cells. (D–F) shows strains with G2/M delays illustrating the three classes of large-budded cell nuclear morphology. (D) Class I (pre-M): overexpression of *TUB2* causes elevation in large-budded mononucleate cells. (E) Class II (early-M): overexpression of *SPC97* accumulates large budded cells with undivided nuclei at the bud necks. (F) Class III (late-M): increased proportions of large-budded cells that had completed nuclear DNA segregation are apparent upon *IME2* overexpression.

The size of the bud relative to the size of the mother cell is the most notable morphological landmark of the cell cycle stages in budding yeast. Bud size was the basis of classical cell cycle screens [7]–[10],[27],[28], allowing the identification of mutants blocked at specific stages of the cell cycle: DNA replication occurs when bud size is small, nuclear division occurs when the bud is about three-fourths the size of the mother cell, and cell separation when the bud is approximately equal in size to the mother cell. In order to independently validate genes in the G1 and G2/M categories using bud size, we measured the ratio of bud size to mother cell size for the 108 ORF strains identified by flow cytometry as having cell cycle defects. Genes in the G1 category caused clearly elevated populations of unbudded cells when overexpressed, and the 20 of 21 genes in the G1 category tested for bud size all exhibited a higher percentage of unbudded cells than control strains (Figure 3A), with 12 being more than 2 standard deviations higher than controls, as shown in Figure 3A. For example, 92% of cells were unbudded and only 2% of cells were large-budded when *TRM5* was overexpressed. In contrast, only 57% of wild type cells were unbudded, and 28% were large-budded (Figure 4B, Table S1). Of 87 strains in the G2/M category, 85 exhibited a higher percentage of large-budded cells than control strains (Figure 3B). For instance, at least 60% of cells had large buds when *TUB2* and *SPC97* were overexpressed (Table S1). Consistent with previous observations, *TRM5*, *TUB2* and *SPC97* are known to cause cell cycle delays when their normal function is perturbed [13],[21],[29],[30]. *SPC97* is an example of the successful recovery of genes known to be important for the cell cycle; it encodes a structural constituent of the spindle pole body, and performs a key role in mitotic spindle formation. 47 strains in the G2/M category had proportions of large-budded cells more than two standard deviations higher than controls, as shown in Figure 3B. Bud size analysis thus provided a useful independent validation of the DNA content observations, with genes validated by both flow cytometry analysis and bud size distributions being the most likely to affect cell cycle progression.

### Subcategorizing Genes Newly Implicated in the Cell Cycle using Drug Sensitivities

One major expected cause of defective cell cycle progression is chromosome instability, especially chromosome loss and non-disjunction. Chromosome loss is characteristic of defects in DNA metabolism, while non-disjunction typically reflects defects in mitotic segregation [15]. To help address which chromosomal functions were primarily affected by the overproduction of the identified ORFs, we examined the strains' sensitivities to hydroxyurea and nocodazole. Hydroxyurea (HU) is an inhibitor of ribonucleotide reductase, an enzyme necessary for DNA synthesis. Nocodazole (NOC) is a microtubule depolymerizing drug that prevents formation of the mitotic spindle. Genes involved in DNA metabolism and the DNA replication checkpoint are often sensitive to HU, whereas genes sensitive to microtubule drugs are often involved with the mitotic checkpoint and mitotic spindle formation [15]. Due to the presence of the spindle checkpoint control, yeast mutants affecting spindle structure normally show cell-cycle arrest in mitosis [31]. We tested the 77 genes potentially newly implicated in the cell cycle for their sensitivity to HU and NOC separately. In the absence of the drugs, we observed all but 4 tested strains (all but *IMG1*, *DHR2*, *GPT2*, and *YGR109W-A*) to show strong growth defects indicative of toxicity of the overexpressed proteins. A semiquantitative score for growth defects, from 0 (no defect) to 3 (strong defect), shows the 77 strains have an average defect of 2.5. Beyond this intrinsic toxicity, we observed 22 strains to be specifically sensitive to NOC, 6 to be specifically sensitive to HU, and 13 strains to show sensitivity to both (Table S4 and Figure S2). As expected, *TUB2* and *PAC2* exhibited the non-disjunction-relevant phenotype, sensitivity to nocodazole but not hydroxyurea; *TUB2* and *PAC2* are required for normal microtubule function and mitotic sister chromatid segregation [21],[24]. We might expect that genes in the same category as *TUB2* and *PAC2* might be directly or indirectly involved in microtubule function or functions related to chromosome segregation, consistent with nearly all (21 of 22) genes having increased sensitivity specifically to NOC arresting at the G2/M phase when overexpressed.

### Functional Analysis of Genes Affecting Cell Cycle Progression when Overexpressed

We examined in more detail the functions for the 108 genes that caused cell cycle defects when overexpressed. Among these genes, 26 are known to be involved in different aspects of cell cycle progression, 21 are essential ORFs, 17 are transcription factors, 20 ORFs are uncharacterized, and 4 are dubious ORFs (Table S2 and Figure 2D). Importantly, of the 26 genes identified in the screen that were previously known for having cell cycle defects, 24 were consistent with the previously observed phenotypes. Of 8 Cdc28p cyclins included in the ORF collection, we recovered 5 (*CLN1*, *CLB2*, *CLB3*, *CLB5*, and *CLB6*). A number of known essential genes cause cell cycle defects when down-regulated [13]; we recovered 67% of these genes in this screen. These observations validate the general quality of the current screen by indicating that cell cycle defects caused by overexpression of these 108 genes do not generally result from random effects of overexpression, but rather the 108 genes are strongly enriched for known regulators of the cell cycle.

We tested to see if the 108 genes were cell cycle regulated or showed obvious expression level biases. They do not appear to be cell cycle regulated, as the set of 108 hits is not significantly enriched for cell-cycle regulated genes as measured by Spellman *et al.* [6] (p>0.05, hypergeometric probability). Analysis of the overexpression levels of the genes show typical induction by 5- to >15-fold over the native expression levels, for proteins of both low and high native levels (Figure S3). We analyzed the distribution of steady state native expression levels of proteins identified in this screen, and do not observe a significant bias in the native levels of the hits; the median expression level of the proteins we identified, measured in rich medium [32], is 2025 copies per cell, versus 2250 copies per cell expected (for all proteins).

We also compared the 108 genes with those previously identified by Sopko *et al.* [16] and Stevenson *et al.* [17] and observe a significant (*p*<0.05, hypergeometric probability) but small overlap, with 15 of the 108 genes observed previously and 93 new to this study (Figure S4). Genes observed in at least two of the three assays are strongly statistically enriched for direct regulators of the cell cycle (e.g., the cylins *CLB3*, *CLB2*, and *CLB5*, and components of the spindle pole body *BIM1*, *TUB2*, *SPC42*, *SPC98*, *KAR1*). Analysis of enriched functions (using Funspec [33]) among genes observed in ≥2 assays reveals the most strongly enriched functions also relate to the cell cycle, with the strongest enrichment observed for the MIPS annotations “cell cycle and DNA processing” (p<10^−7^), “cell cycle” (p<10^−6^), and “mitotic cell cycle and cell cycle control” (p<10^−6^).

In the next two sections, we describe the G1 and G2/M genes in more detail.

### Genes Causing G2/M Delays

The 87 G2/M genes showed dramatic enrichment in cell cycle-related Gene Ontology (GO) biological process annotations, including regulation of CDK activity [GO:0000079] (*p*<9×10^−7^), microtubule-based process [GO:0007017] (*p*<2×10^−6^), cell cycle [GO:0007049] (*p*<4×10^−6^), cytoskeleton organization and biogenesis [GO:0007010] (*p*<8×10^−6^), microtubule cytoskeleton organization and biogenesis [GO:0000226] (*p*<8×10^−6^), G2/M transition of mitotic cell cycle [GO:0000086] (*p*<5×10^−5^), DNA replication and chromosome cycle [GO:0000067] (*p*<5×10^−5^), and related processes. These genes include *CLB2*, *CLB3*, *CLB5*, *CDC31*, *KAR1*, *SPC97*, *PAC2*, *TUB2*, *NIP100*, *SLK19*, *ASK1*, *AME1*, *MAD2*, and *ACT1*, which have direct roles in regulating the G2/M transition and related processes such as microtubule nucleation, chromosome segregation, and mitotic spindle checkpoint control. Additionally, 7 genes identified in previous large-scale studies [13],[16],[17] (*SPO13*, *SEC17*, *MYO2*, *PRP31*, *ARF1*, *TFG2*, and *SHE1*), although not directly involved in mitotic cell cycle control, were also observed in this study. Of 63 genes newly identified in this screen (3 were not tested for growth phenotype), 56 caused slow growth upon induction and the overexpression of 21 genes lead to specific sensitivity to nocodazole.

**Figure 5:**
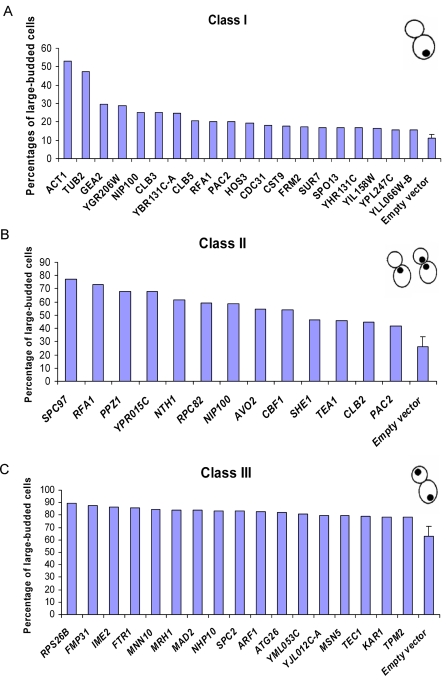
Genes in the G2/M category can be categorized based upon nuclear DNA staining in large-budded cells. (A) Category I (20 genes): an undivided nucleus in one cell body. (B) Category II (13 genes): undivided nuclei in bud neck. (C) Category III (17 genes): two divided nuclei separated to two cell bodies.

In order to better classify the genes by the nature of their overexpression defects, *i.e*., as to whether the cells exhibited M phase arrest or whether chromosome segregation defects led to G2/M arrest, 3 classes of nuclear morphology were assigned based on the patterns of DNA staining, as shown in Figure 4 D–F: an undivided nucleus in one cell body (class I, pre-M), an undivided nucleus in the bud neck (class II, early-M), and divided nuclei in two cell bodies (class III, late-M) [17]. In control strains, 60% of the cells exhibited class III nuclear morphology, with chromosomes in these cells successfully segregated, while only 11% of cells showed class I morphology, and 26% of cells class II morphology. We observed 20 ORF strains to have significantly elevated percentages (95% confidence level) of cells with class I morphology, 13 ORF strains with class II, and 17 ORF strains with class III (Figure 5). Among the 33 genes in the Class I and II, 9 have direct roles in regulating G2/M transition (*CLB2*, *CLB3* and *CLB5*), or related important events in the mitotic cell division phase (*ACT1*, *TUB2*, *NIP100*, *PAC2*, *CDC31*, *SPC97*). For example, Spc97p is a component of the microtubule-nucleating Tub4p (gamma-tubulin) complex and overproduction of *SPC97* causes microtubule defects, which in turn gives rise to a failure of chromosome segregation and a early M phase arrest (Figure 5B) [29]. We therefore reasoned that 24 newly implicated Class I and II genes causing a similar phenotype to that of *SPC97* might play direct or indirect roles in chromosome segregation, especially for genes whose overexpression also leads to hyper sensitivity to nocodazole (*GEA2*, *RFA1, HOS3*, *YPR015C*, *AVO2*, *CBF1*, *SHE1*, and *TEA1;* Figure S2). We characterized two of these genes, *RFA1* and *YPR015C*, in more detail.

### Overexpression of *YPR015C* Results in Mitotic Instability and Activates the DNA Damage Checkpoint

**Figure 6:**
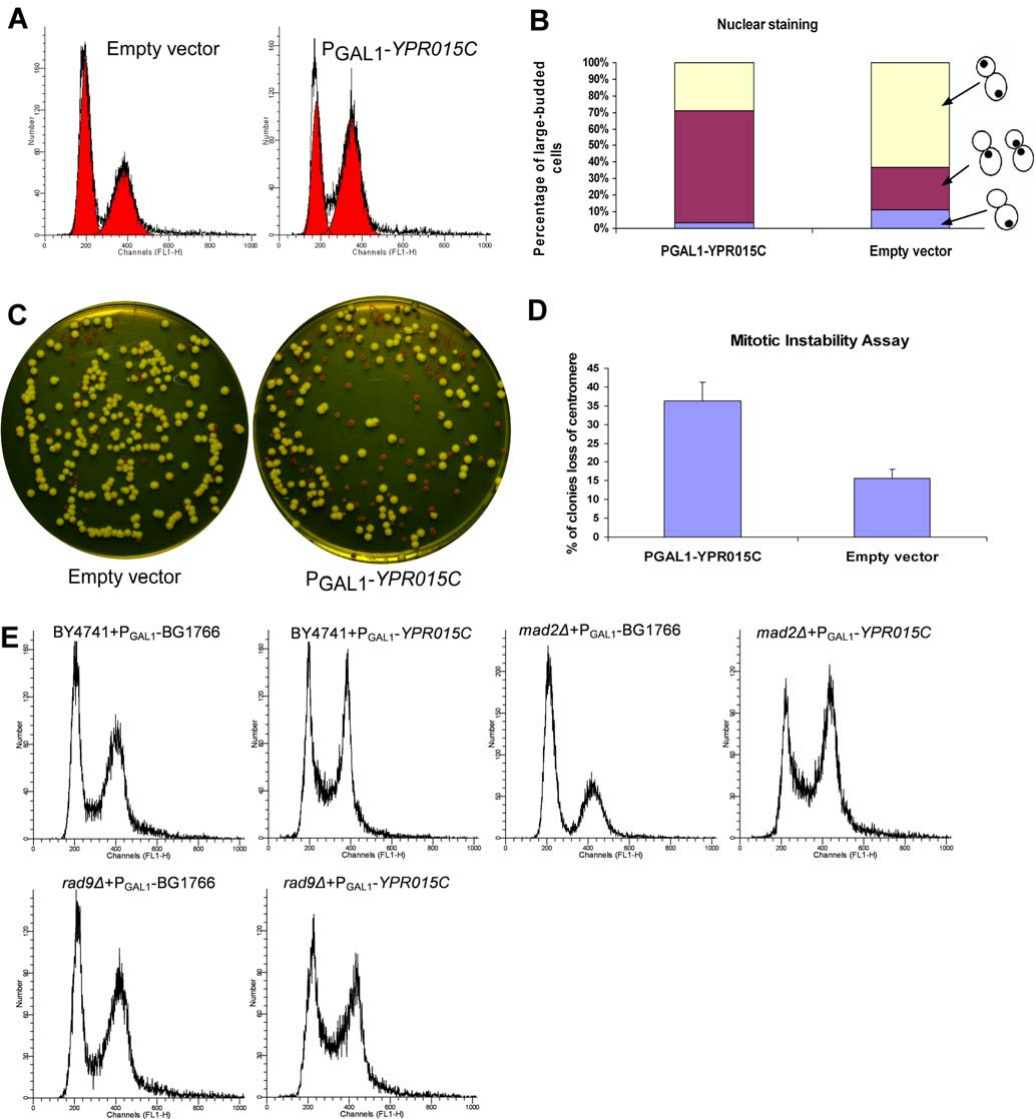
Overexpression of *YPR015C* results in mitotic instability and activates the DNA damage checkpoint. (A) Flow cytometry histograms of empty vector control strain and P_GAL1_-*YPR015C* strain show the G2/M delay phenotype upon overexpression *YPR015C*. (B) Summary of the results from analysis of nuclear DNA staining. P_GAL1_-*YPR015C* showed a higher percentage of large-budded cells with undivided nuclei at the bud neck than the empty vector control strain. (C) An assay of mitotic instability using a reporter plasmid (pRS412::ADE2 [cir+]) shows that *YPR015C* overexpression increases mitotic instability, indicated by an increase in red colonies (signifying loss of the centromere-containing plasmid) relative to white colonies (correctly carrying the plasmid). Quantitation of this trend (D) reveals the P_GAL1_-*YPR015C* strain to have about twice the rate of centromere loss than that of the empty vector control strain. (E) Flow cytometry indicates that deletion of *RAD9* suppressed the G2/M delay caused by overexpression of *YPR015C*, while deletion of *MAD2* did not suppress the G2/M delays caused by overexpression of *YPR015C*, indicating that the G2/M delay requires *RAD9*, and thus the DNA damage checkpoint.

*YPR015C* encodes an uncharacterized putative transcription factor known to exhibit synthetic lethality with and be functionally linked to *CTF4* [34],[35]; both genes have zinc finger motifs. *CTF4* encodes a chromatin-associated protein required for sister chromatid cohesion, which in turn regulates high-fidelity chromosome segregation (Hanna et al., 2001). Deletion of CTF4 increases chromosome instability and causes early mitotic delay [36]–[38]. We observe overexpression of *YPR015C* to give rise to a very similar phenotype to deletion of *CTF4*. *YPR015C* overexpression causes hyper sensitivity to nocodazole and slight sensitivity to hydroxyurea (Figure S2), and an elevated population of large-budded cells with the nucleus in the bud neck (Figure 6B). In order to test whether the overexpression of *YPR015C* also leads to chromosome instability, we overexpressed *YPR015C* in the strain Cry1 (MAT a *ade2-1, ura3-1, leu2-3, 112, trp1, his3-11*) carrying the low copy centromere-containing plasmid pRS412::ADE2 [cir+]. Overexpression of *YPR015C* doubled the rate of loss of centromere plasmids: 36% in the *YPR015C* overexpressing strain *vs.* 16% in the wild type control strain, indicating chromosome instability and mis-segregation.

Bud size and nuclear morphology indicated that cells arrested in early mitosis phase when *YPR015C* was overexpressed (Figure 6B). To test whether the early mitotic delay caused by the overexpression of *YPR015C* is due to activation of the DNA damage checkpoint or the spindle assembly checkpoint, we overexpressed *YPR015C* in the background of *rad9*Δ or *mad2*Δ mutants in which the DNA damage or spindle assembly checkpoints were removed, respectively. Cell cycle progression in these mutants was measured by DNA content analysis of galactose-induced cultures (Figure 6E). We observed that the *YPR015C*-induced early mitotic delay was dependent on the DNA damage checkpoint and not the spindle assembly checkpoint, in contrast to the early mitotic delay caused by deletion of *CTF4,* which is dependent on the spindle checkpoint [37]. Interestingly, three ribonucleotide reductases (*RNR2*, *RNR3*, *RNR4*) are the most significantly up-regulated genes following overexpression of *YPR015C* [39], and these three ribonucleotide reductases are regulated by the DNA replication and DNA damage checkpoint pathways [40]. Since transcriptional response, DNA replication, DNA repair, and chromosome condensation are the major chromatin restructuring events in cohesin operation [37], it appears that overexpression of *YPR015C* may interfere with chromosome cohesion, inducing defects in mitotic chromosome segregation *via* a different mechanism than *CTF4*.

### Overexpression of *RFA1* Induces Chromosome Segregation and Spindle Defects

**Figure 7:**
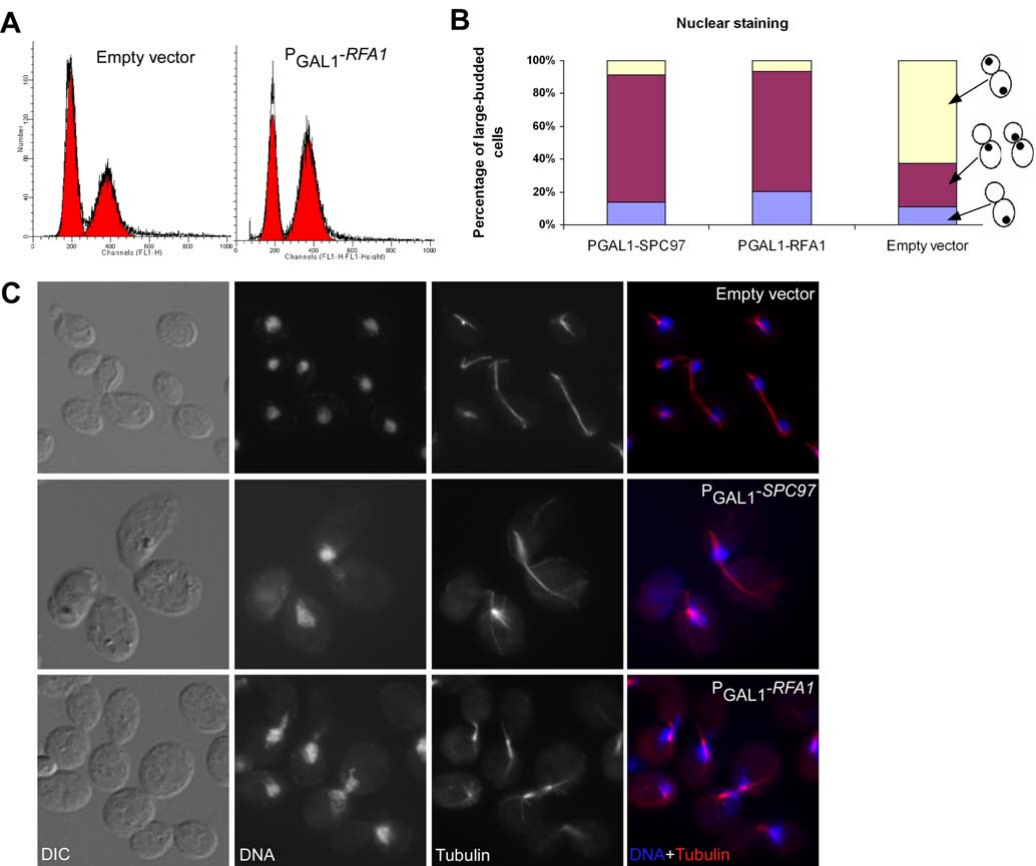
Overexpression of *RFA1* causes chromosomal segregation and spindle defects. (A) The G2/M delay phenotype upon overexpression of *RFA1* is apparent in flow cytometry histograms of the empty vector control strain and the P_GAL1_-*RFA1* strain. (B) Quantitation of cell microscopy results following nuclear DNA staining indicates that P_GAL1_-*SPC97* and P_GAL1_-*RFA1* strains exhibit considerably higher percentages of large-budded cells with undivided nuclei than the empty vector control strain. (C) Log-phase cultures of the wild type control strain and cells carrying P_GAL1_-*SPC97* or P_GAL1_-*RFA1* were fixed in formaldehyde and stained to visualize DNA (by DAPI) and microtubules (by immunofluorescence). The cells carrying the empty vector correctly showed a long anaphase spindle, with nuclei successfully segregated into two cell bodies. Overexpression of either *SPC97* or *RFA1* resulted in a failure of chromosome segregation; the spindle morphology of P_GAL1_-*RFA1* cells is distinct from that of P_GAL1_-*SPC97* cells, with shorter mitotic spindles poorly aligned with the division axis.

*RFA1* is another gene involved in DNA replication whose overexpression leads to G2/M delay. The Rfa1p protein is a subunit of the heterotrimeric replication protein A (RPA), which is involved in DNA replication, repair, and the DNA damage checkpoint [41],[42]. RFA1 is essential for yeast viability, an *RFA1* null mutant is inviable [18]. However, several point mutations of *RFA1* caused accumulation of large-budded [43] or dumb-bell shaped cells with a single nucleus in the bud neck [42] at the nonpermissive temperature and had defects in DNA replication and DNA repair [42]–[45]. We observe ∼73% of large-budded cells of the *RFA1* overexpression strain showed a butterfly-shaped nucleus in their bud necks, similar to phenotype of *SPC97* overexpression (*i.e.*, asymmetric chromosome segregation) and fewer than 10% of large-budded cells had chromosomes segregated into two cell bodies (Figure 7B), suggestive of chromosome mis-segregation. In contrast, 63% of large-budded cells of the parental control strain had the chromosomes successfully segregated into two cell bodies.

Furthermore, we observed that the *RFA1* overexpression strain had short mitotic spindles, with spindle pole bodies not clearly attached to the nucleus (Figure 7C, lower row). This defect is distinct from the spindle morphology caused by overexpression of *SPC97* (Figure 7C, middle row); Spc97p is a component of the microtubule-nucleating Tub4p (gamma-tubulin) complex and is involved in spindle pole body separation and mitotic spindle formation. Cells either carrying point mutations [29] or overexpressing *SPC97* (Figure 7C, middle row) had short spindles and elongated cytoplasmic microtubules, but the spindle pole appeared normally attached to the nucleus. Given that Rfa1p is a single-stranded DNA binding protein involved in DNA replication, it seems likely that overexpression of *RFA1* disrupts DNA replication and leads to the observed spindle morphology defects, giving rise to the observed early mitotic delay. Such a role would also be consistent with the observation that DNA replication proteins can act as cohesion proteins and play important roles in regulating spindle integrity and maintaining the tension on chromosomes exerted by spindle microtubules [37],[46],[47].

### Genes Causing G1 Delays

While the strains arresting in G2/M phase were strongly enriched for cell cycle associated functions, diverse mechanisms are known to induce G1 arrests [16],[17]. This diversity was reflected in the enrichment of GO biological process annotations among the G1 arresting ORFs: no pathway was enriched at *p*<0.001 when calculated by the method of [33], consistent with previous overexpression studies [16],[17]. When calculated as in [25], the strongest enrichment consisted of negative regulators of transcription from RNA polymerase II promoters (GO:0000122; *p*<4×10^−4^).

Among the 21 genes inducing G1 delays, 6 (29%) are uncharacterized or dubious ORFs. The only functional information available for *YOR131C* and *YDR493W* is localization: *YOR131C* is localized in the nucleus and cytoplasm, and *YDR493W* is localized in mitochondria [18],[19],[48]. Our data further associate these two genes with cell cycle progression, either directly or indirectly. Tma64p is another protein of unknown function, previously identified in a mass spectrometry-based proteomic screen of yeast ribosomal complexes [49]. Tma64p associates with ribosomes, has a RNA binding domain and interacts with Rps4bp, a component of the small (40S) ribosomal subunit [50]. Moreover, it has been suggested that there might be a strong connection between ribosomal biogenesis and G1 transit [11],[13]. Therefore, the G1 delay caused by overexpression of *TMA64* may suggest a role in ribosomal biogenesis.

The weak enrichment observed for transcriptional regulators derives from 4 transcription factors involved in responding to environmental stress that were observed in the G1 category. Three are transcriptional repressors (*MIG3*, *NCB2*, and *SKO1*), and the fourth (*GAT4)* is unclear as to mode of action. We observed unusual cellular morphology upon overexpression of *SKO1*, and examined this repressor in more detail.

### Overexpression of *SKO1* Activates the Pheromone Response Pathway

**Figure 8:**
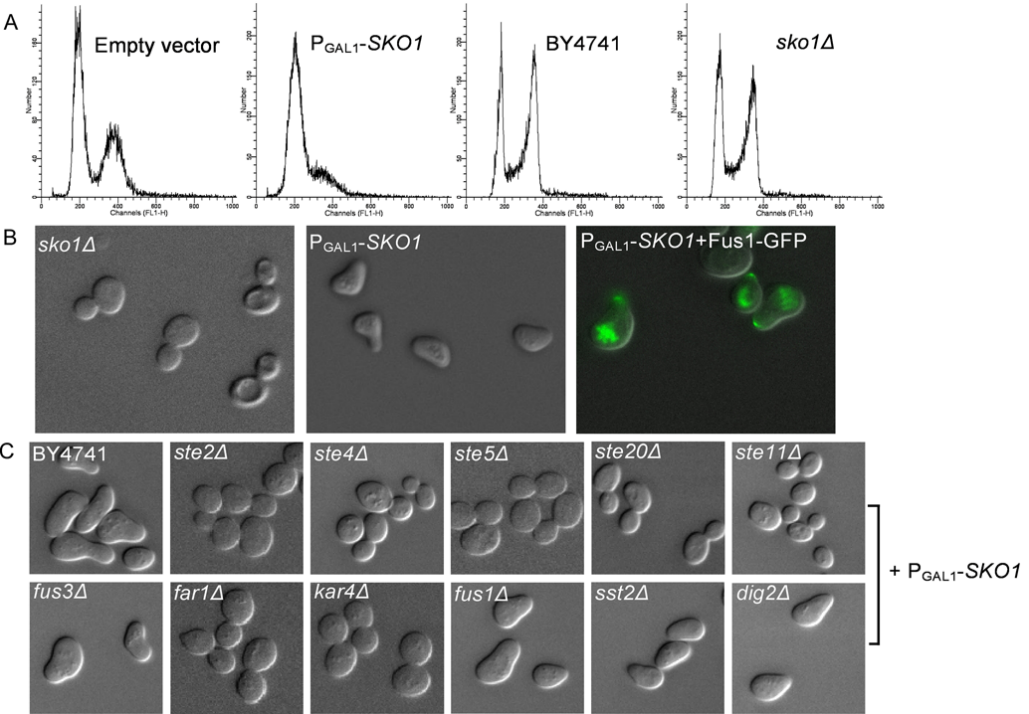
Overexpression of *SKO1* activates the pheromone response pathway. (A) shows flow cytometry analysis of DNA content for the empty vector control strain, P_GAL1_-*SKO1* strain, and the *sko1*Δ strain with its corresponding control strain. Overproduction of *SKO1* causes a strong arrest at the G1 phase (78% of P_GAL1_-*SKO1* cells accumulated at the G1 phase *vs.* 58% of control cells at the G1 phase). In contrast, there was no obvious G1 arrest in the *sko1*Δ strain. (B) While the *sko1*Δ strain exhibits a typical yeast cell morphology, cells from the P_GAL1_-*SKO1* strain resemble yeast cells presented with mating pheromone (shmoos). Overexpression of *SKO1* in cells expressing a green-fluorescent protein-tagged version of the mating projection marker Fus1 induces Fus1-GFP localization to the tip of the projection (shown as an overlay of the GFP channel on the DIC image), consistent with *SKO1* overexpression inducing shmooing. (C) *SKO1* induces shmooing when overexpressed in the deletion strains *fus1*Δ, *fus3*Δ, *sst2*Δ, and *dig2*Δ, as well as in the corresponding parental strain (BY4741), but not when overexpressed in the deletion strains *ste2*Δ, *ste4*Δ, *ste5*Δ, *ste20*Δ, *ste11*Δ, *far1*Δ, and *kar4*Δ, indicating that the latter genes are required for *SKO1*-induced shmoo formation.

We observed overproduction of *SKO1* to strongly inhibit cell growth and arrest cells at the G1 phase (Figure 8A). Bud size analysis showed that 90% of cells had no bud when *SKO1* was overexpressed (Table S1). *SKO1* is a basic leucine zipper (bZIP) transcription factor of the ATF/CREB family, involved in osmotic and oxidative stress responses. The Sko1p protein forms a complex with Tup1p and Ssn6p to both activate and repress transcription [51]–[53]. Surprisingly, overproduction of *SKO1* resulted in formation of shmoos, cell morphology changes that are normally seen in mating yeast in response to mating pheromone (Figure 8B). We reasoned that the elevated expression of *SKO1* might activate the pheromone response pathway either directly or indirectly, causing shmoo formation and a mating-associated G1 arrest.

Since Fus1p is a marker protein induced during shmoo formation that localizes to the shmoo tip when the pheromone response pathway is activated [54], we tested *SKO1* activation of the pheromone response pathway by examining the localization of Fus1p when *SKO1* was overexpressed. We transformed P_GAL1_-*SKO1* plasmids into a MATa strain in which *FUS1* was C-terminally tagged with green fluorescent protein (GFP) [19]. Upon *SKO1* overexpression, Fus1-GFP localized to the shmoo tip (Figure 8B), resembling its localization pattern upon alpha factor treatment, demonstrating that the morphological changes are accompanied by general activation of the mating pathway, thus explaining the G1 cell cycle arrest phenotype of the *SKO1* ORF strains.

To further explore which genes involved in the pheromone MAP kinase pathway were activated by the overexpression of *SKO1*, we performed cDNA microarray profiling and found that the activated genes were highly enriched in pheromone response and mating genes. Significantly upregulated genes (*p*<0.01) included *MFA1*, *STE2, BAR1, FAR1, FUS1, KAR4, FIG1, FIG2, GIC2, PRM4, PRM5, PRM8, AGA1,* and *AGA2*, as listed in Table S5. To establish direct genetic interactions between *SKO1* and pheromone response pathway, we overexpressed *SKO1* strains in *ste2*Δ, *ste4*Δ, *ste20*Δ, *ste11*Δ, *ste5*Δ, *kar4*Δ, *fus3*Δ, *far1*Δ, *fus1*Δ, *sst2*Δ, and *dig2*Δ strains, and examined whether or not *SKO1* overexpression induced shmoo formation in these deletion strains. We did not observe *SKO1*-induced shmoo formation in *ste2*Δ, *ste4*Δ, *ste20*Δ, *ste11*Δ, *ste5*Δ, *kar4*Δ, and *far1*Δ strains (Figure 8C), indicating that these genes are required for shmoo induction by *SKO1* overexpression. *FUS3* is functionally compensated by *KSS1*, *FUS1* is downstream of the pheromone response signal transduction pathway, *SST2* and *DIG2* are inhibitors in the pathway; deletion of these genes affects neither pheromone nor *SKO1*-dependent shmoo induction. The observed effects of *SKO1* overexpression on cell cycle progression thus appear to be indirect, activating the pheromone response pathway in a manner dependent upon the pheromone receptor (*STE2*) and MAP kinase signal transduction pathway, and this activation in turn results in G1 arrest through the normal mating pheromone-mediated pathway.

### Overexpression Phenotypes Are Generally Distinct from Loss-of-Function Phenotypes

**Figure 9:**
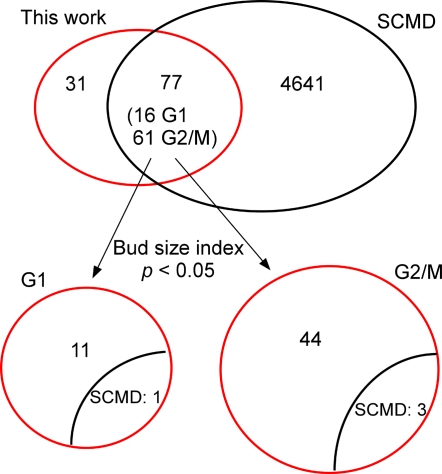
Overexpression phenotypes are generally distinct from loss-of-function phenotypes. Of 108 genes causing cell cycle defects when overexpressed, quantitative cell morphology information for 77 of the corresponding deletion mutants was available in the *Saccharomyces cerevisiae* Morphology Database (SCMD), with 16 genes in the G1 category and 61 in the G2/M category. Considering only those ORF strains whose bud size index differs from control strains with *p*<0.05, 12 genes caused significantly higher percentages of cells without buds than control strains when overexpressed. Of these 12 genes, only 1 gene led to significantly high proportions of unbudded cells when deleted. For G2/M genes, 47 genes caused significantly elevated percentages of cells with large buds upon overexpression; only 3 of them also lead to significantly high populations of large-budded cells when deleted. Thus, the large majority of overexpression phenotypes are not mirrored by the corresponding deletion strains, raising the likelihood for overexpression phenotypes to have arisen through gain-of-function mechanisms.

Overexpression of a normal gene product can result in gain-of-function, but may also mimic loss-of-function phenotypes [16], such as in cases where precise levels of a protein are required, with either too much or too little equally disruptive. In order to systematically assess the extent of these phenomena amongst the phenotypes of the overexpression strains, we took advantage of quantitative cell morphology data (bud count data) for deletion strains collected in the *Saccharomyces cerevisiae* Morphology Database **(**SCMD) [55] and compared them to our quantitative bud count data. Of 108 genes from this screen, 77 also appear in SCMD (21 essential genes and 10 additional genes are not included in SCMD) (Figure 9). We selected genes from our screen with significantly elevated populations (*p*<0.05) of unbudded cells or large-budded cells. In the G1 category, there were 12 strains from our screen whose percentages of cells without buds were significantly higher than that of wild type. Of these 12 G1 genes, only one also led to a significantly elevated population of unbudded cells when deleted, as measured by SCMD. Therefore, our rough estimate is that 11/12 (92%) of genes in the G1 category exhibit an overexpression phenotype distinct from the loss-of-function phenotype, at least as measured with regard to proportions of unbudded cells. Similarly, 44 (94%) genes in the G2/M category caused a significantly elevated proportion of large-budded cells when overexpressed but not when deleted, versus 3 that resembled the loss-of-function phenotype (Figure 9, Table S3). Thus, the majority of the overexpressed genes in this paper appear to exhibit a phenotype distinct from the loss-of-function case, supporting the previously hypothesized notion that gain-of-function may be common amongst the overexpression phenotypes [16].

*SKO1* appears to represent such an example of a gain-of-function leading to differences between the overexpression phenotype and the corresponding deletion phenotype. When overexpressed, *SKO1,* which encodes a transcription repressor responsive to salt and osmotic stresses, activates the pheromone response pathway and leads to a strong G1 arrest, but the deletion of *SKO1* has no detectable arrest or mating phenotype (Figure 8B). Moreover, transcriptional profiling of cells overexpressing *SKO1* revealed that genes involved in the pheromone response pathway are significantly upregulated. However, genes involved in the pheromone response pathway do not appear to be regulated by *SKO1* under normal culture conditions, at least as measured by chromatin-immunoprecipitation of *SKO1* [56]. Therefore, our results suggest that *SKO1* regulates genes in the pheromone response pathway through a gain-of-function mechanism, e.g., such as by enabling binding to a cryptic or lower affinity promoter when overexpressed.

### Conclusions

In this paper, we describe a near-saturating screen for yeast genes whose overexpression causes cell cycle delays and which are thus likely to function in cell cycle progression. We individually examined the effects of overexpression on cell cycle progression for each of ∼5,556 yeast ORFs, and report the 108 genes with the most significant and reproducible cell cycle defects. 82 of these genes have not been reported in previous large-scale screens [13],[16],[17], probably due to different overexpression conditions and strain backgrounds, false positives in large-scale screens [11], or more likely, false negatives, e.g., such as might derive from variable 2 micron plasmid copy numbers [57] increasing phenotypic variability and thus allowing cell cycle defects to escape detection. Our analysis thus complements previous screens. These results lay the foundation for future experiments to elucidate the precise roles of these genes in cell cycle progression, such as the mechanisms of *RFA1* and *YPR015C*. Overexpression screens such as we have described here provide complementary information to loss-of-function studies and therefore offer new opportunities for discovery of genetic interactions, such as by systematically testing the overexpression plasmids in deletion strains to screen for phenotype suppression or synthetic interactions. Finally, since overexpression is an efficient technique in human cell culture and since regulation of cell proliferation is an important aspect of studying human diseases, we anticipate that a similar effort to this work in human cell lines could accelerate our understanding of cell cycle control in mammalian systems and help to further clarify the many connections between cell cycle control and cancer.

## Supporting Information

Figure S1 Flow cytometry histograms of 108 ORF overexpression strains causing cell cycle defects upon induction.
(0.44 MB PDF)

Figure S2 77 of 82 ORF strains not previously known to show cell cycle defects upon induction were tested for drug sensitive growth phenotypes.
(10.62 MB PDF)

Figure S3 Protein expression is significantly induced in overexpression strains, even for proteins expressed natively at high levels.
(0.14 MB PDF)

Figure S4 Overlap of identified genes with previous large-scale studies.
(0.15 MB PDF)

Table S1 108 yeast ORFs causing cell cycle defects when overexpressed.
(0.03 MB PDF)

Table S2 Summary of 108 strains with cell cycle defects.
(0.01 MB PDF)

Table S3 Comparison between over-expression and loss-of-function phenotypes.
(0.01 MB PDF)

Table S4 Genes whose overexpression induces slow growth, drug sensitivity.
(0.01 MB PDF)

Table S5 Genes upregulated following overexpression of *SKO1*.
(0.02 MB PDF)

Table S6 Over-expression strains appearing diploid or 3C.
(0.04 MB PDF)

## References

[1] Hartwell LH (1974). Saccharomyces cerevisiae cell cycle.. Bacteriol Rev.

[2] Mendenhall MD, Hodge AE (1998). Regulation of Cdc28 cyclin-dependent protein kinase activity during the cell cycle of the yeast Saccharomyces cerevisiae.. Microbiol Mol Biol Rev.

[3] Schafer KA (1998). The cell cycle: a review.. Vet Pathol.

[4] Cho RJ, Campbell MJ, Winzeler EA, Steinmetz L, Conway A (1998). A genome-wide transcriptional analysis of the mitotic cell cycle.. Mol Cell.

[5] de Lichtenberg U, Jensen LJ, Fausboll A, Jensen TS, Bork P (2005). Comparison of computational methods for the identification of cell cycle-regulated genes.. Bioinformatics.

[6] Spellman PT, Sherlock G, Zhang MQ, Iyer VR, Anders K (1998). Comprehensive identification of cell cycle-regulated genes of the yeast Saccharomyces cerevisiae by microarray hybridization.. Mol Biol Cell.

[7] Hartwell LH (1971). Genetic control of the cell division cycle in yeast. II. Genes controlling DNA replication and its initiation.. J Mol Biol.

[8] Hartwell LH (1971). Genetic control of the cell division cycle in yeast. IV. Genes controlling bud emergence and cytokinesis.. Exp Cell Res.

[9] Hartwell LH (1973). Three additional genes required for deoxyribonucleic acid synthesis in Saccharomyces cerevisiae.. J Bacteriol.

[10] Hartwell LH, Culotti J, Reid B (1970). Genetic control of the cell-division cycle in yeast. I. Detection of mutants.. Proc Natl Acad Sci U S A.

[11] Bjorklund M, Taipale M, Varjosalo M, Saharinen J, Lahdenpera J (2006). Identification of pathways regulating cell size and cell-cycle progression by RNAi.. Nature.

[12] Kittler R, Pelletier L, Heninger AK, Slabicki M, Theis M (2007). Genome-scale RNAi profiling of cell division in human tissue culture cells.. Nat Cell Biol.

[13] Yu L, Castillo LP, Mnaimneh S, Hughes TR, Brown GW (2006). A survey of essential gene function in the yeast cell division cycle.. Mol Biol Cell.

[14] Gelperin DM, White MA, Wilkinson ML, Kon Y, Kung LA (2005). Biochemical and genetic analysis of the yeast proteome with a movable ORF collection.. Genes Dev.

[15] Ouspenski, Elledge SJ, Brinkley BR (1999). New yeast genes important for chromosome integrity and segregation identified by dosage effects on genome stability.. Nucleic Acids Res.

[16] Sopko R, Huang D, Preston N, Chua G, Papp B (2006). Mapping pathways and phenotypes by systematic gene overexpression.. Mol Cell.

[17] Stevenson LF, Kennedy BK, Harlow E (2001). A large-scale overexpression screen in Saccharomyces cerevisiae identifies previously uncharacterized cell cycle genes.. Proc Natl Acad Sci U S A.

[18] Giaever G, Chu AM, Ni L, Connelly C, Riles L (2002). Functional profiling of the Saccharomyces cerevisiae genome.. Nature.

[19] Huh WK, Falvo JV, Gerke LC, Carroll AS, Howson RW (2003). Global analysis of protein localization in budding yeast.. Nature.

[20] Haase SB, Reed SI (2002). Improved flow cytometric analysis of the budding yeast cell cycle.. Cell Cycle.

[21] Huffaker TC, Thomas JH, Botstein D (1988). Diverse effects of beta-tubulin mutations on microtubule formation and function.. J Cell Biol.

[22] Schwartz K, Richards K, Botstein D (1997). BIM1 encodes a microtubule-binding protein in yeast.. Mol Biol Cell.

[23] Sobel SG, Snyder M (1995). A highly divergent gamma-tubulin gene is essential for cell growth and proper microtubule organization in Saccharomyces cerevisiae.. J Cell Biol.

[24] Hoyt MA, Macke JP, Roberts BT, Geiser JR (1997). Saccharomyces cerevisiae PAC2 functions with CIN1, 2 and 4 in a pathway leading to normal microtubule stability.. Genetics.

[25] Hu Z, Killion PJ, Iyer VR (2007). Genetic reconstruction of a functional transcriptional regulatory network.. Nat Genet.

[26] Hughes TR, Marton MJ, Jones AR, Roberts CJ, Stoughton R (2000). Functional discovery via a compendium of expression profiles.. Cell.

[27] Culotti J, Hartwell LH (1971). Genetic control of the cell division cycle in yeast. 3. Seven genes controlling nuclear division.. Exp Cell Res.

[28] Moir D, Stewart SE, Osmond BC, Botstein D (1982). Cold-sensitive cell-division-cycle mutants of yeast: isolation, properties, and pseudoreversion studies.. Genetics.

[29] Knop M, Pereira G, Geissler S, Grein K, Schiebel E (1997). The spindle pole body component Spc97p interacts with the gamma-tubulin of Saccharomyces cerevisiae and functions in microtubule organization and spindle pole body duplication.. Embo J.

[30] Fitch I, Dahmann C, Surana U, Amon A, Nasmyth K (1992). Characterization of four B-type cyclin genes of the budding yeast Saccharomyces cerevisiae.. Mol Biol Cell.

[31] Winsor B, Schiebel E (1997). Review: an overview of the Saccharomyces cerevisiae microtubule and microfilament cytoskeleton.. Yeast.

[32] Ghaemmaghami S, Huh WK, Bower K, Howson RW, Belle A (2003). Global analysis of protein expression in yeast.. Nature.

[33] Robinson MD, Grigull J, Mohammad N, Hughes TR (2002). FunSpec: a web-based cluster interpreter for yeast.. BMC Bioinformatics.

[34] Lee I, Li Z, Marcotte EM (2007). An Improved, Bias-Reduced Probabilistic Functional Gene Network of Baker's Yeast, Saccharomyces cerevisiae.. PLoS ONE.

[35] Tong AH, Lesage G, Bader GD, Ding H, Xu H (2004). Global mapping of the yeast genetic interaction network.. Science.

[36] Spencer F, Gerring SL, Connelly C, Hieter P (1990). Mitotic chromosome transmission fidelity mutants in Saccharomyces cerevisiae.. Genetics.

[37] Hanna JS, Kroll ES, Lundblad V, Spencer FA (2001). Saccharomyces cerevisiae CTF18 and CTF4 are required for sister chromatid cohesion.. Mol Cell Biol.

[38] Miles J, Formosa T (1992). Evidence that POB1, a Saccharomyces cerevisiae protein that binds to DNA polymerase alpha, acts in DNA metabolism in vivo.. Mol Cell Biol.

[39] Chua G, Morris QD, Sopko R, Robinson MD, Ryan O (2006). Identifying transcription factor functions and targets by phenotypic activation.. Proc Natl Acad Sci U S A.

[40] Yao R, Zhang Z, An X, Bucci B, Perlstein DL (2003). Subcellular localization of yeast ribonucleotide reductase regulated by the DNA replication and damage checkpoint pathways.. Proc Natl Acad Sci U S A.

[41] Majka J, Binz SK, Wold MS, Burgers PM (2006). Replication protein A directs loading of the DNA damage checkpoint clamp to 5′-DNA junctions.. J Biol Chem.

[42] Longhese MP, Plevani P, Lucchini G (1994). Replication factor A is required in vivo for DNA replication, repair, and recombination.. Mol Cell Biol.

[43] Umezu K, Sugawara N, Chen C, Haber JE, Kolodner RD (1998). Genetic analysis of yeast RPA1 reveals its multiple functions in DNA metabolism.. Genetics.

[44] Longhese MP, Neecke H, Paciotti V, Lucchini G, Plevani P (1996). The 70 kDa subunit of replication protein A is required for the G1/S and intra-S DNA damage checkpoints in budding yeast.. Nucleic Acids Res.

[45] Firmenich AA, Elias-Arnanz M, Berg P (1995). A novel allele of Saccharomyces cerevisiae RFA1 that is deficient in recombination and repair and suppressible by RAD52.. Mol Cell Biol.

[46] Skibbens RV, Corson LB, Koshland D, Hieter P (1999). Ctf7p is essential for sister chromatid cohesion and links mitotic chromosome structure to the DNA replication machinery.. Genes Dev.

[47] Wang Z, Castano IB, De Las Penas A, Adams C, Christman MF (2000). Pol kappa: A DNA polymerase required for sister chromatid cohesion.. Science.

[48] Reinders J, Zahedi RP, Pfanner N, Meisinger C, Sickmann A (2006). Toward the complete yeast mitochondrial proteome: multidimensional separation techniques for mitochondrial proteomics.. J Proteome Res.

[49] Fleischer TC, Weaver CM, McAfee KJ, Jennings JL, Link AJ (2006). Systematic identification and functional screens of uncharacterized proteins associated with eukaryotic ribosomal complexes.. Genes Dev.

[50] Krogan NJ, Peng WT, Cagney G, Robinson MD, Haw R (2004). High-definition macromolecular composition of yeast RNA-processing complexes.. Mol Cell.

[51] Nehlin JO, Carlberg M, Ronne H (1992). Yeast SKO1 gene encodes a bZIP protein that binds to the CRE motif and acts as a repressor of transcription.. Nucleic Acids Res.

[52] Pascual-Ahuir A, Posas F, Serrano R, Proft M (2001). Multiple levels of control regulate the yeast cAMP-response element-binding protein repressor Sko1p in response to stress.. J Biol Chem.

[53] Pascual-Ahuir A, Serrano R, Proft M (2001). The Sko1p repressor and Gcn4p activator antagonistically modulate stress-regulated transcription in Saccharomyces cerevisiae.. Mol Cell Biol.

[54] Trueheart J, Boeke JD, Fink GR (1987). Two genes required for cell fusion during yeast conjugation: evidence for a pheromone-induced surface protein.. Mol Cell Biol.

[55] Saito TL, Ohtani M, Sawai H, Sano F, Saka A (2004). SCMD: Saccharomyces cerevisiae Morphological Database.. Nucleic Acids Res 32 Database issue.

[56] Lee TI, Rinaldi NJ, Robert F, Odom DT, Bar-Joseph Z (2002). Transcriptional regulatory networks in Saccharomyces cerevisiae.. Science.

[57] Futcher AB, Cox BS (1983). Maintenance of the 2 microns circle plasmid in populations of Saccharomyces cerevisiae.. J Bacteriol.

