# Supplementary material for: Mechanisms of Cell Cycle Control Revealed by a Systematic and Quantitative Overexpression Screen in S. cerevisiae
Source: PLoS Genet. 2008 Jul 11;4(7):e1000120. doi: 10.1371/journal.pgen.1000120 (PMC2438615; doi:10.1371/journal.pgen.1000120)
Supplement: Figure S1 — Flow cytometry histograms of 108 ORF overexpression strains causing cell cycle defects upon induction. (0.44 MB PDF) [file pgen.1000120.s001.pdf]

Supplemental Figure 1: Flow cytometry histograms of 108 ORF overexpression strains causing cell cycle defects upon induction. Fluorescence intensity (DNA content) is indicated on the x-axis; the number of cells with a given intensity is plotted on the y-axis. G1-arresting cells are presented first, followed by G2/M & controls.

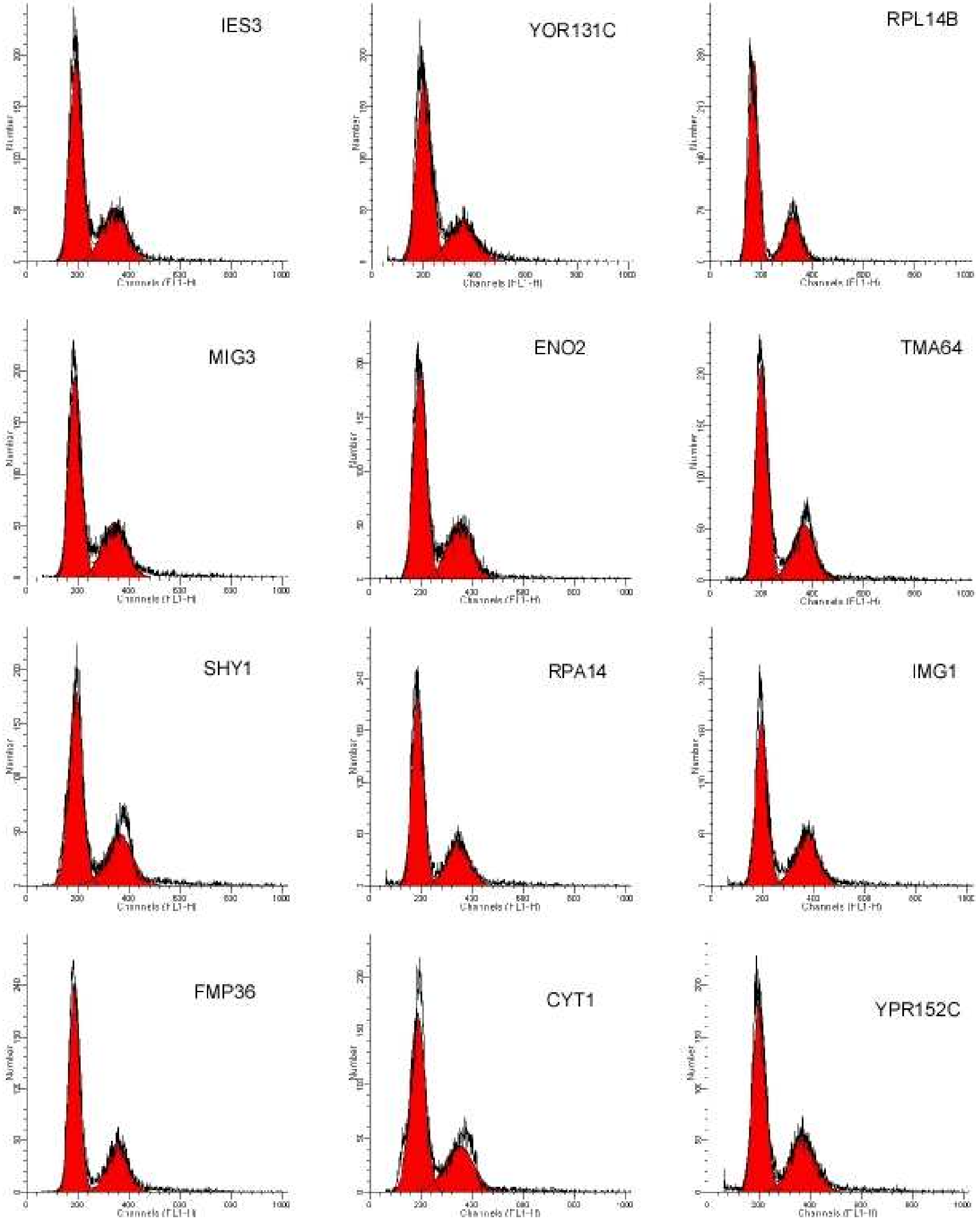
IES3
YOR131C
RPL14B
MIG3
ENO2
TMA64
SHY1
RPA14
IMG1
FMP36
CYT1
YPR152C

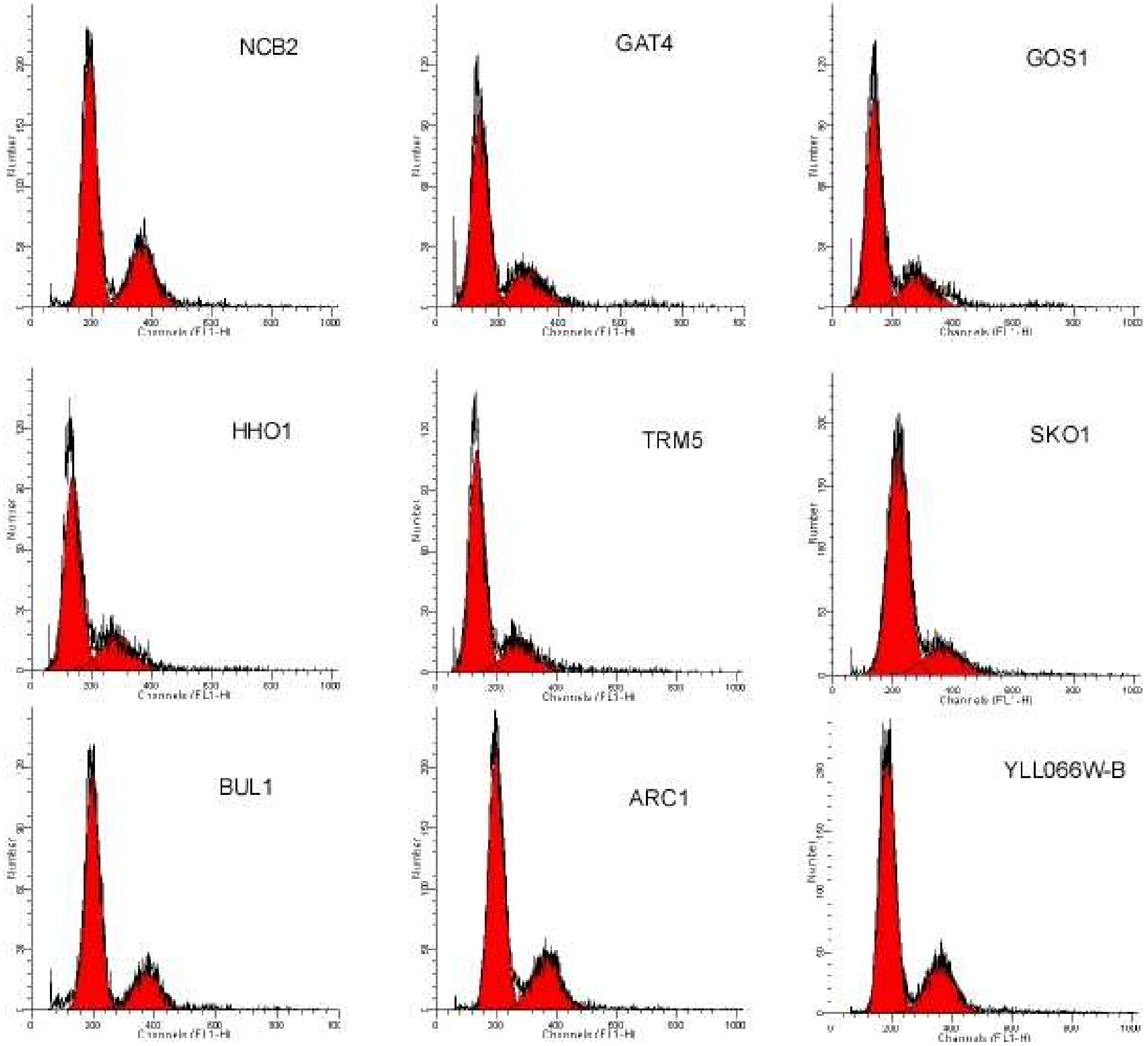
NCB2
GAT4
GOS1
HHO1
TRM5
SKO1
BUL1
ARC1
YLL066W-B

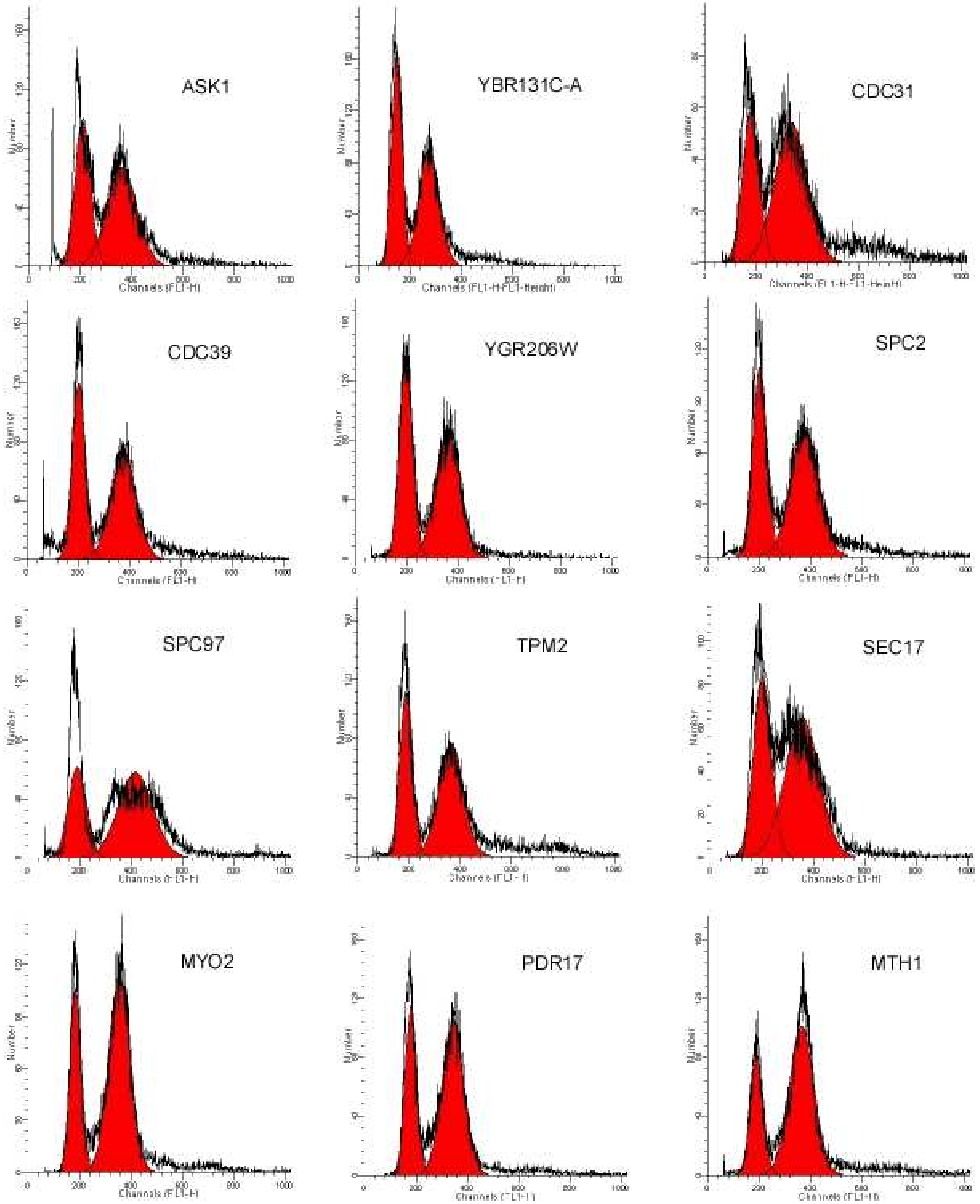
ASK1
YBR131C-A
CDC31
CDC39
YGR206W
SPC2
SPC97
TPM2
SEC17
MYO2
PDR17
MTH1

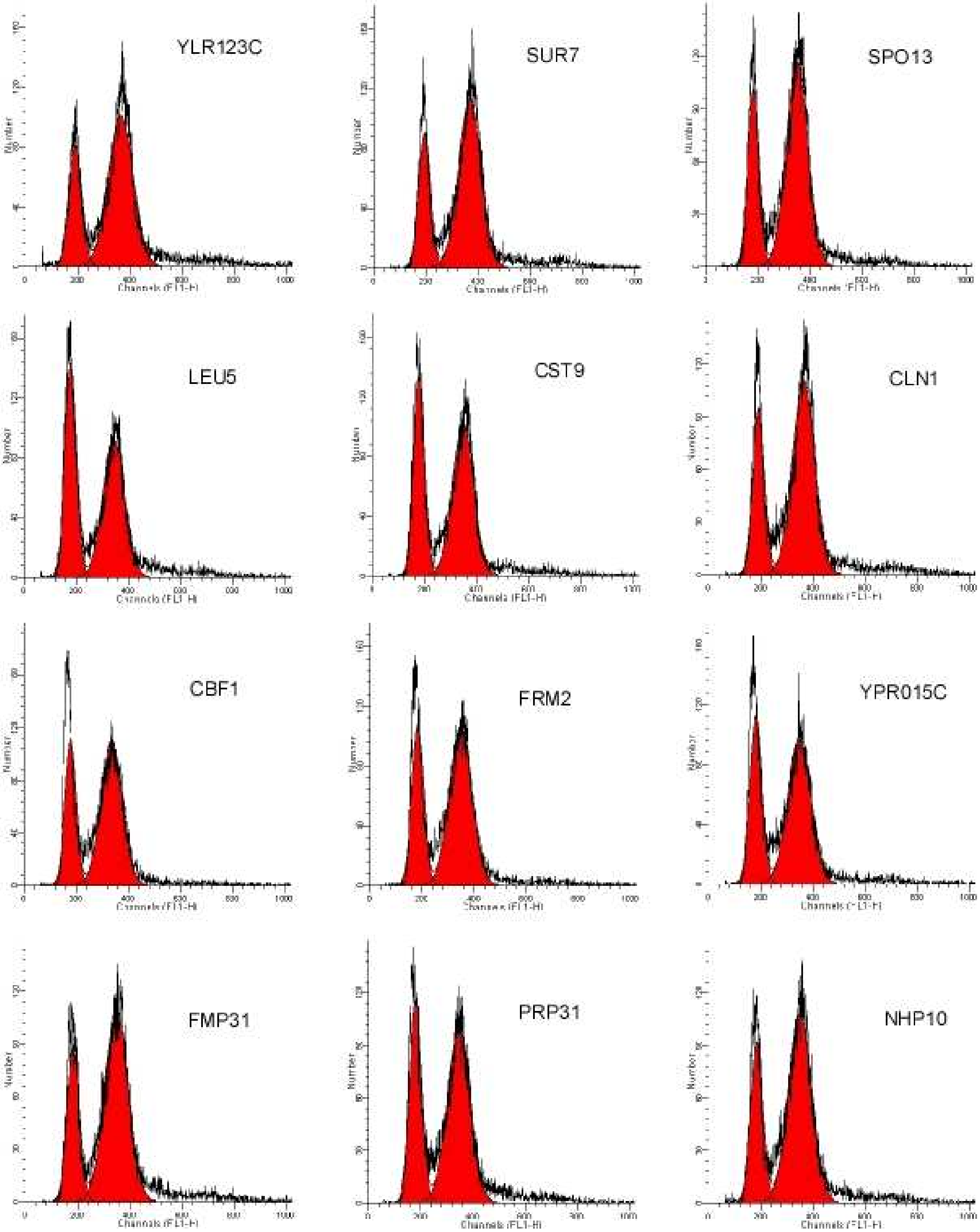
YLR123C
SUR7
SPO13
LEU5
CST9
CLN1
CBF1
FRM2
YPR015C
FMP31
PRP31
NHP10

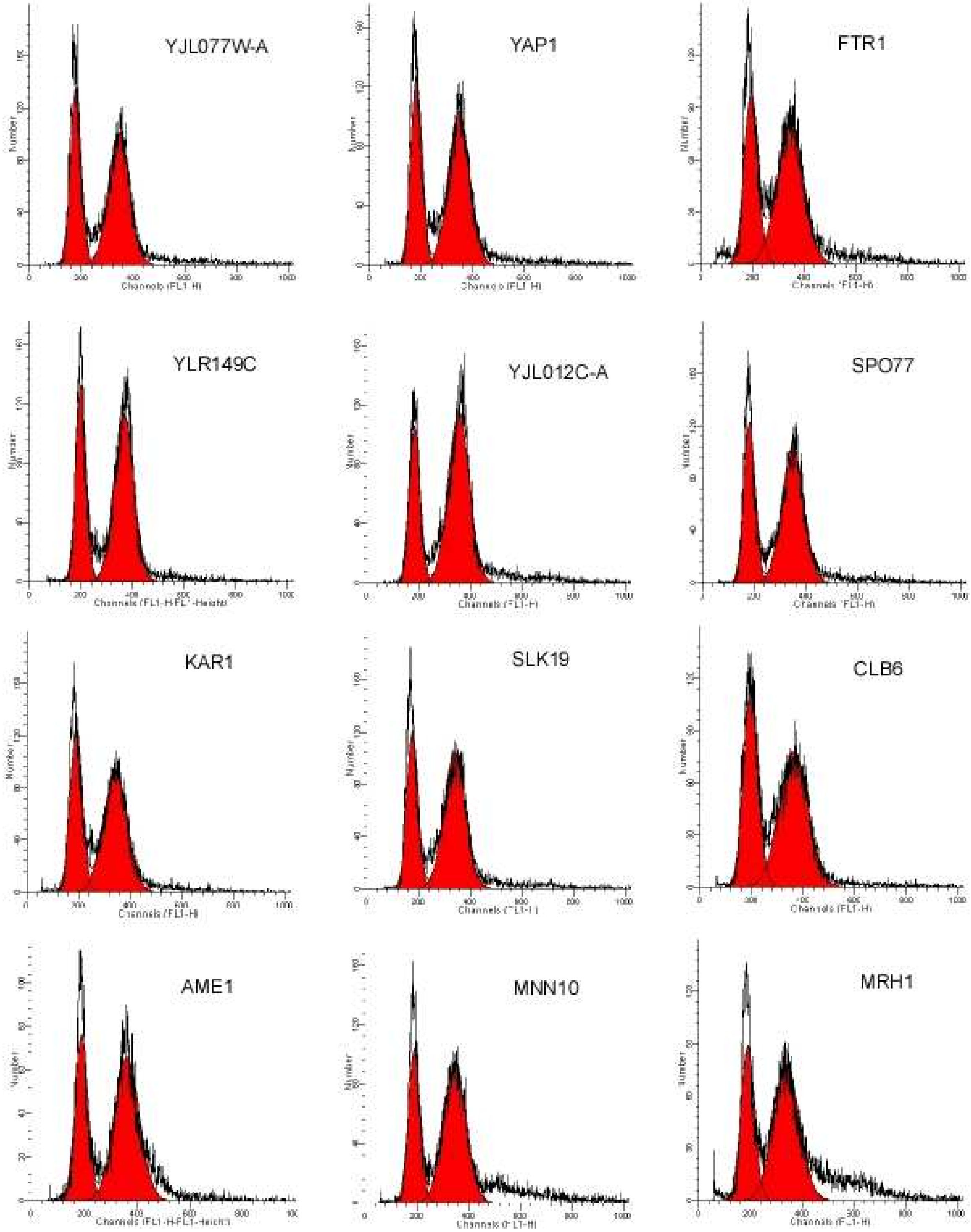
YJL077W-A
YAP1
FTR1
YLR149C
YJL012C-A
SPO77
KAR1
SLK19
CLB6
AME1
MNN10
MRH1

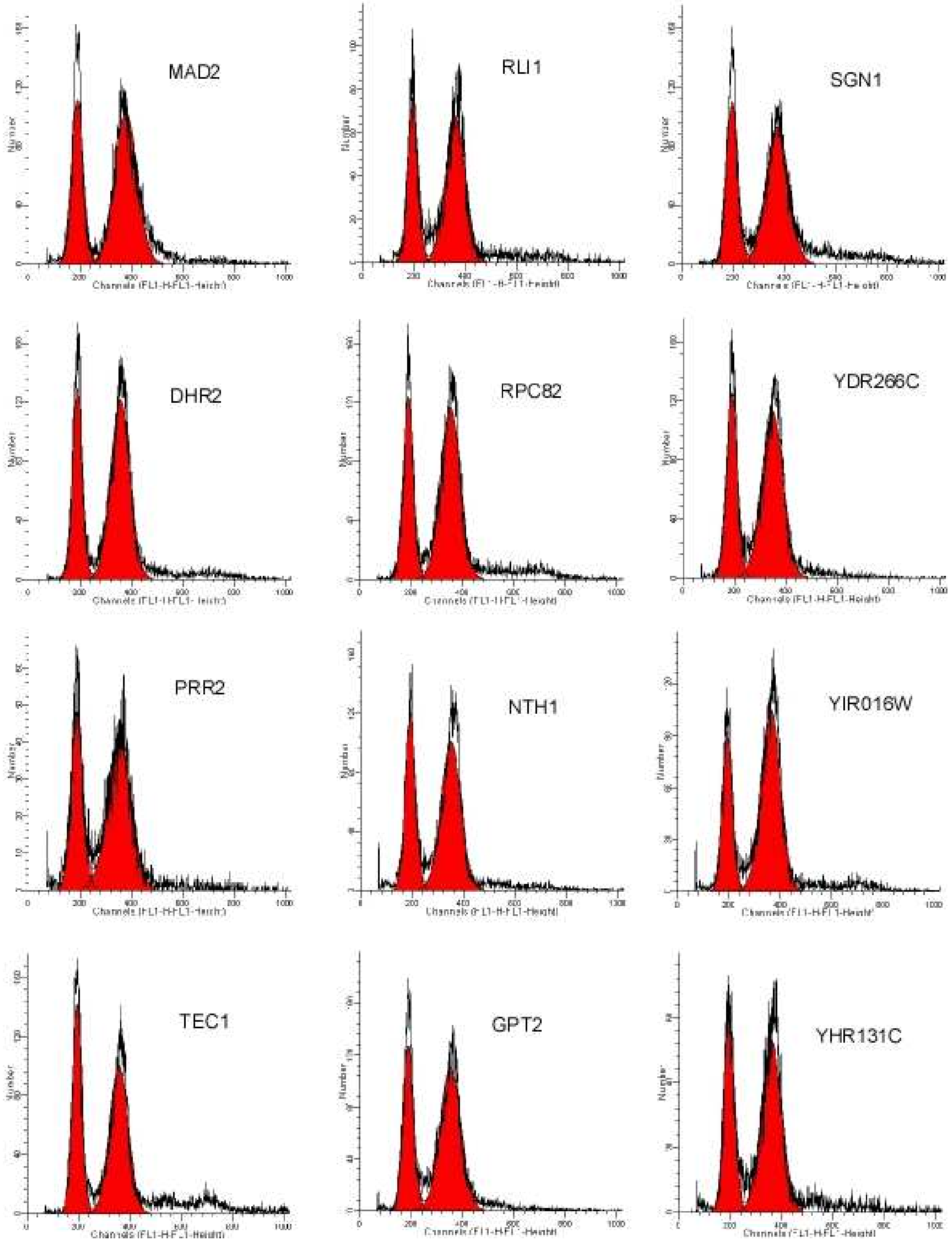
MAD2
RLI1
SGN1
DHR2
RPC82
YDR266C
PRR2
NTH1
YIR016W
TEC1
GPT2
YHR131C

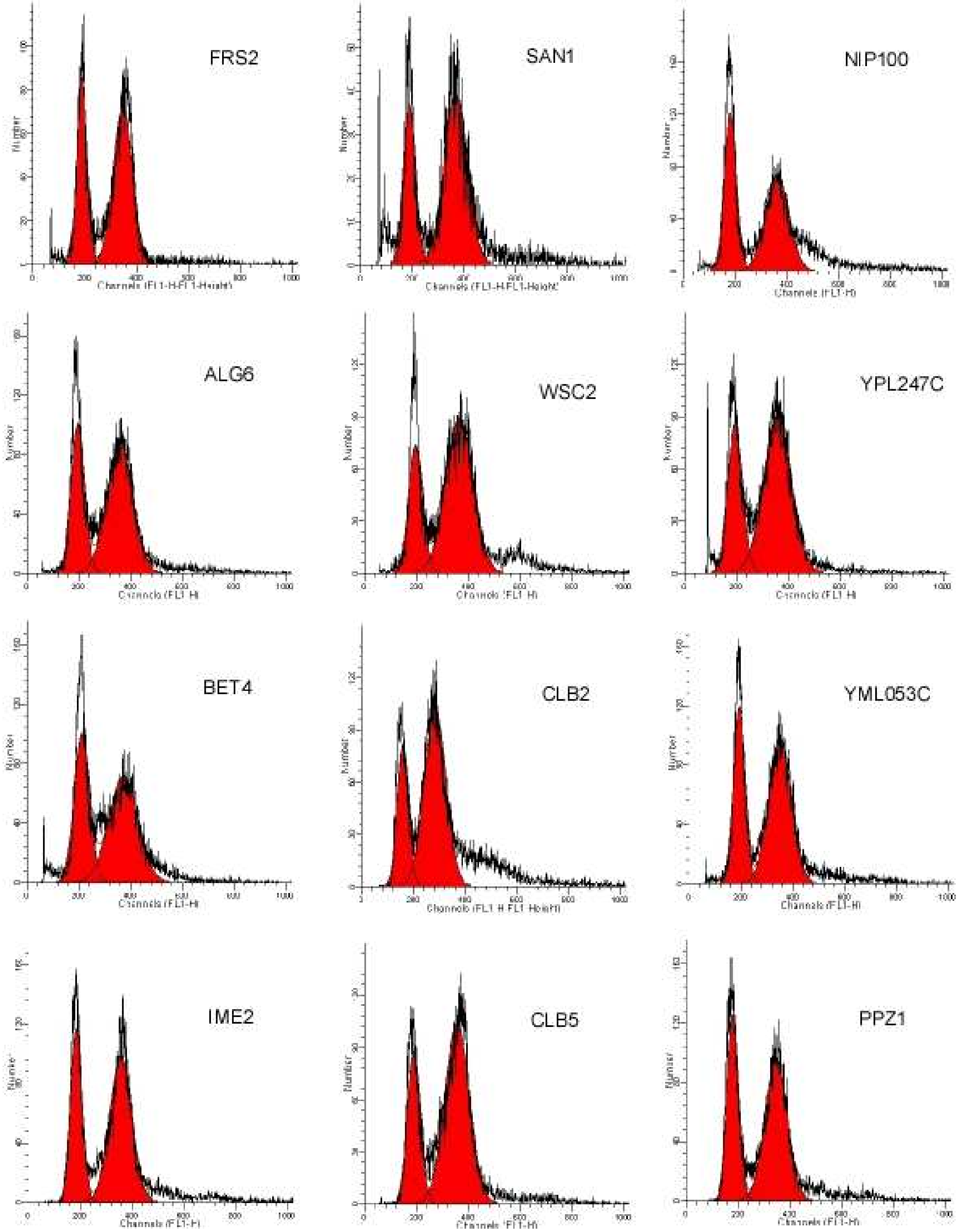
FRS2
SAN1
NIP100
ALG6
WSC2
YPL247C
BET4
CLB2
YML053C
IME2
CLB5
PPZ1

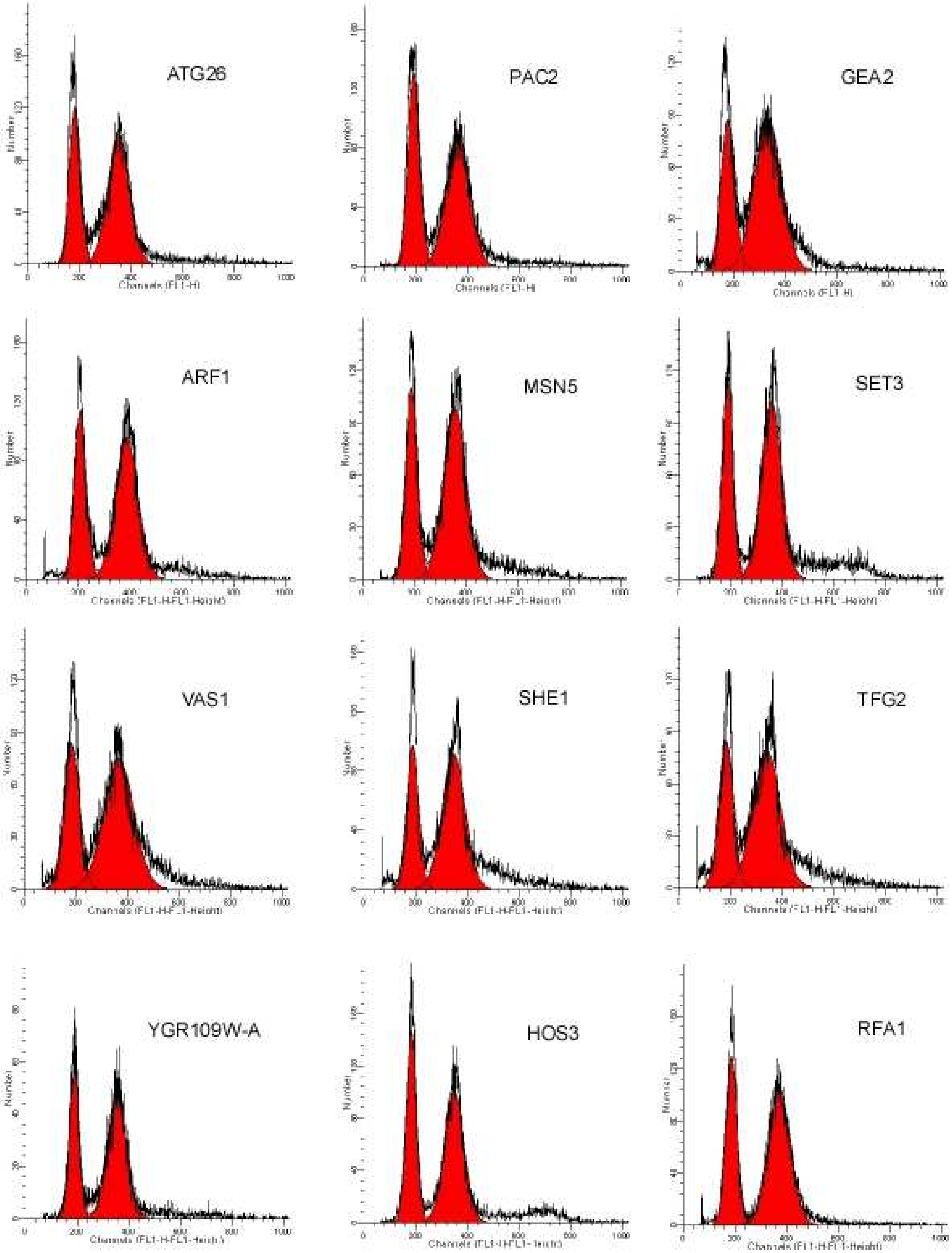
ATG26
PAC2
GEA2
ARF1
MSN5
SET3
VAS1
SHE1
TFG2
YGR109W-A
HOS3
RFA1

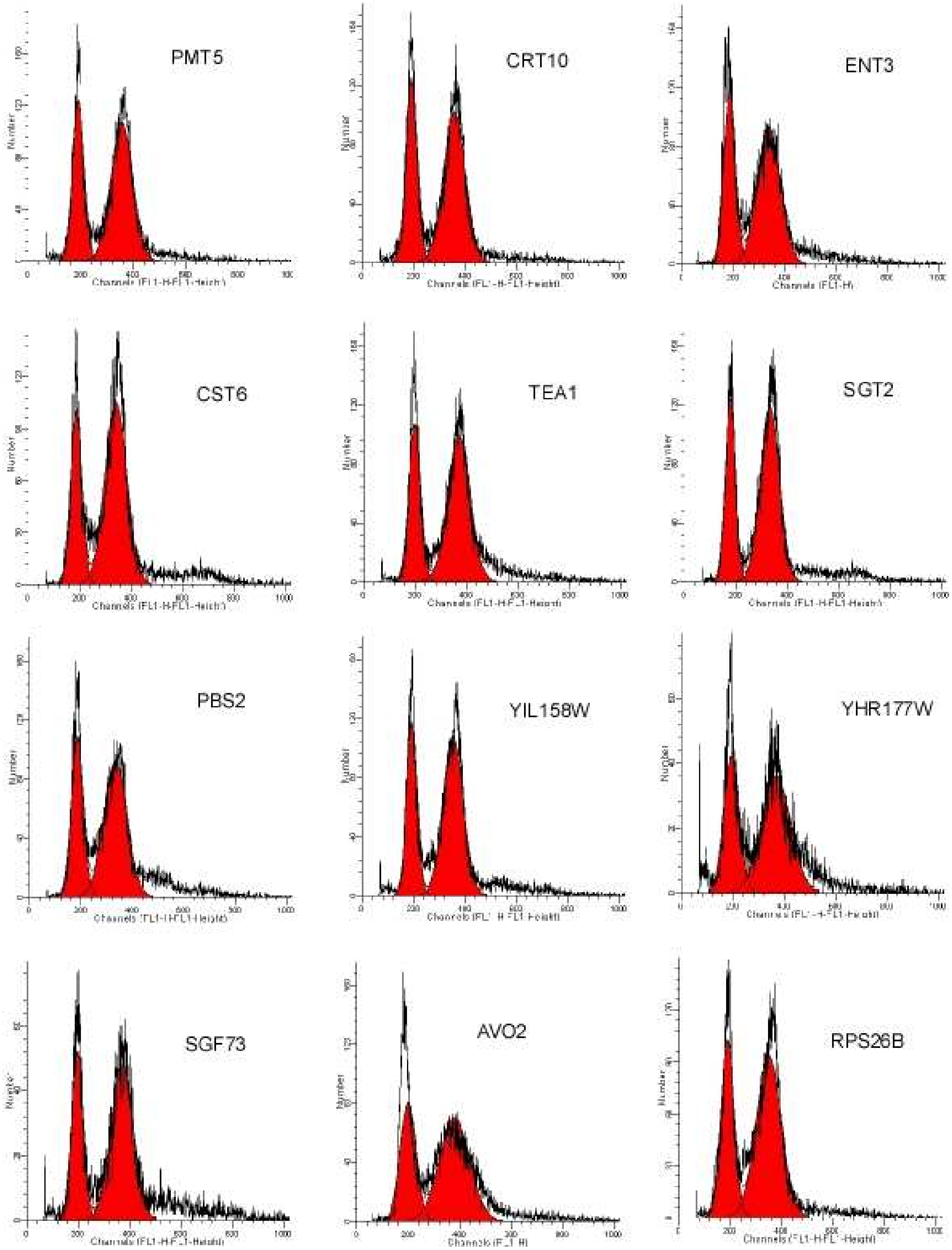
PMT5
CRT10
ENT3
CST6
TEA1
SGT2
PBS2
YIL158W
YHR177W
SGF73
AVO2
RPS26B

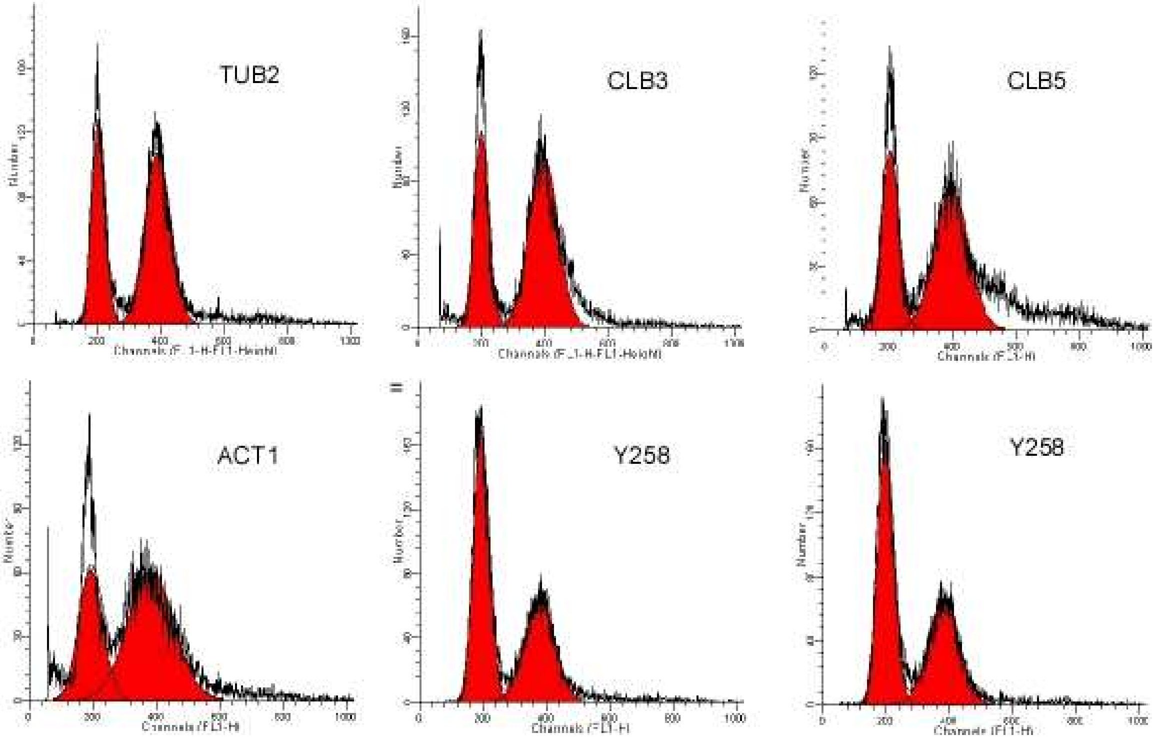
TUB2
CLB3
CLB5
ACT1
Y258
Y258
