# Supplementary material for: Mechanisms of Cell Cycle Control Revealed by a Systematic and Quantitative Overexpression Screen in S. cerevisiae
Source: PLoS Genet. 2008 Jul 11;4(7):e1000120. doi: 10.1371/journal.pgen.1000120 (PMC2438615; doi:10.1371/journal.pgen.1000120)
Supplement: Figure S2 — 77 of 82 ORF strains not previously known to show cell cycle defects upon induction were tested for drug sensitive growth phenotypes. (10.62 MB PDF) [file pgen.1000120.s002.pdf]

Supplemental Figure 2: 77 of the 82 ORF strains not previously known to show cell cycle defects upon induction were tested for drug sensitive growth phenotypes. Cells containing the control plasmid BG1766 and the indicated cell cycle genes from the screen were spotted onto SC-URA, 2% galactose plates or SC-URA, 2% galactose plates containing 50µM hydroxyurea (HU) or 15µg/ml nocodazole (NOC), and photographed after growth at 30 ^{o}C as described in Materials and Methods.

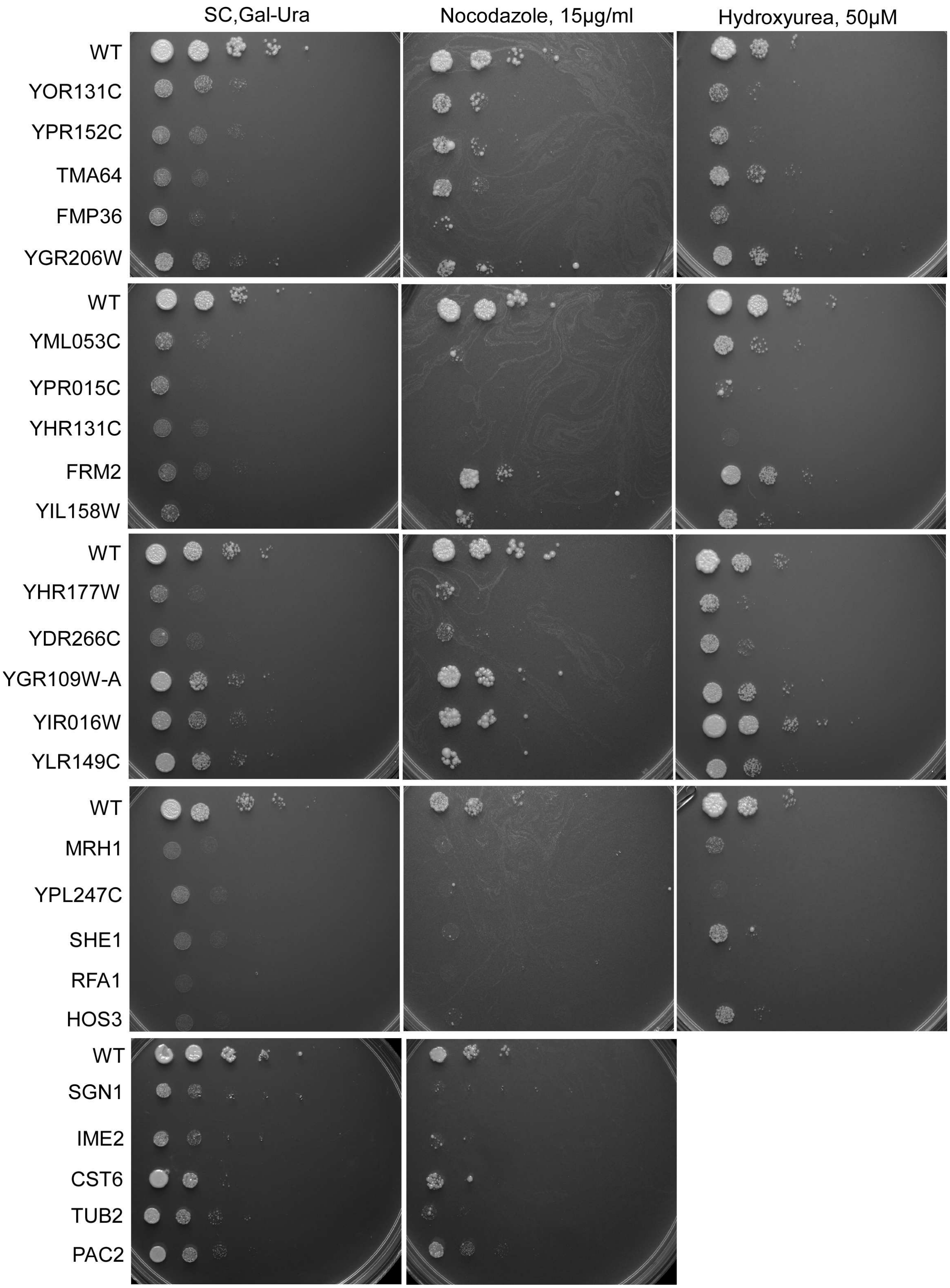
SC,Gal-Ura
Nocodazole, 15μg/ml
Hydroxyurea, 50μM
WT
YOR131C
YPR152C
TMA64
FMP36
YGR206W
WT
YML053C
YPR015C
YHR131C
FRM2
YIL158W
WT
YHR177W
YDR266C
YGR109W-A
YIR016W
YLR149C
WT
MRH1
YPL247C
SHE1
RFA1
HOS3
WT
SGN1
IME2
CST6
TUB2
PAC2

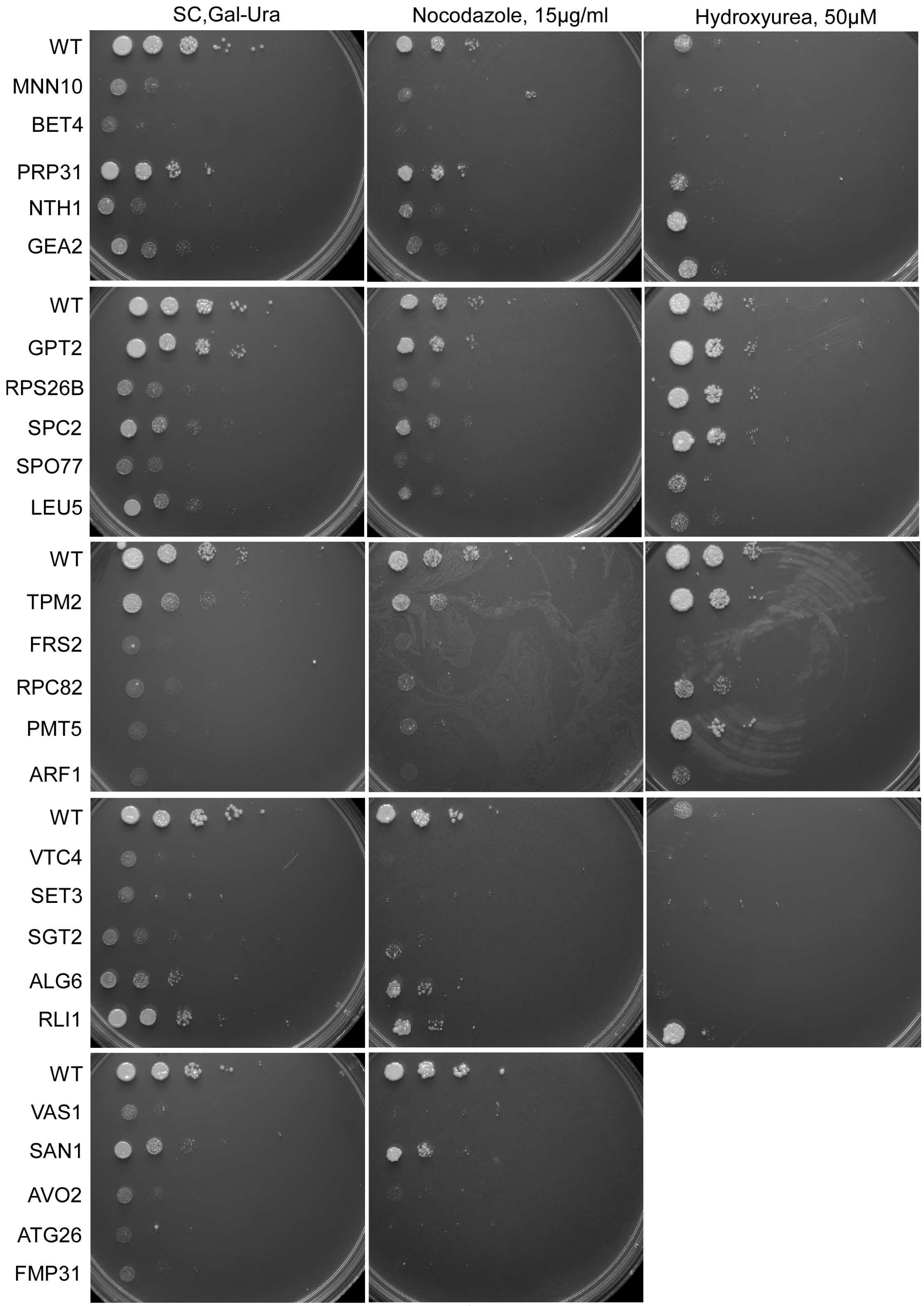
SC,Gal-Ura
Nocodazole, 15µg/ml
Hydroxyurea, 50µM
WT
MNN10
BET4
PRP31
NTH1
GEA2
WT
GPT2
RPS26B
SPC2
SPO77
LEU5
WT
TPM2
FRS2
RPC82
PMT5
ARF1
WT
VTC4
SET3
SGT2
ALG6
RLI1
WT
VAS1
SAN1
AVO2
ATG26
FMP31

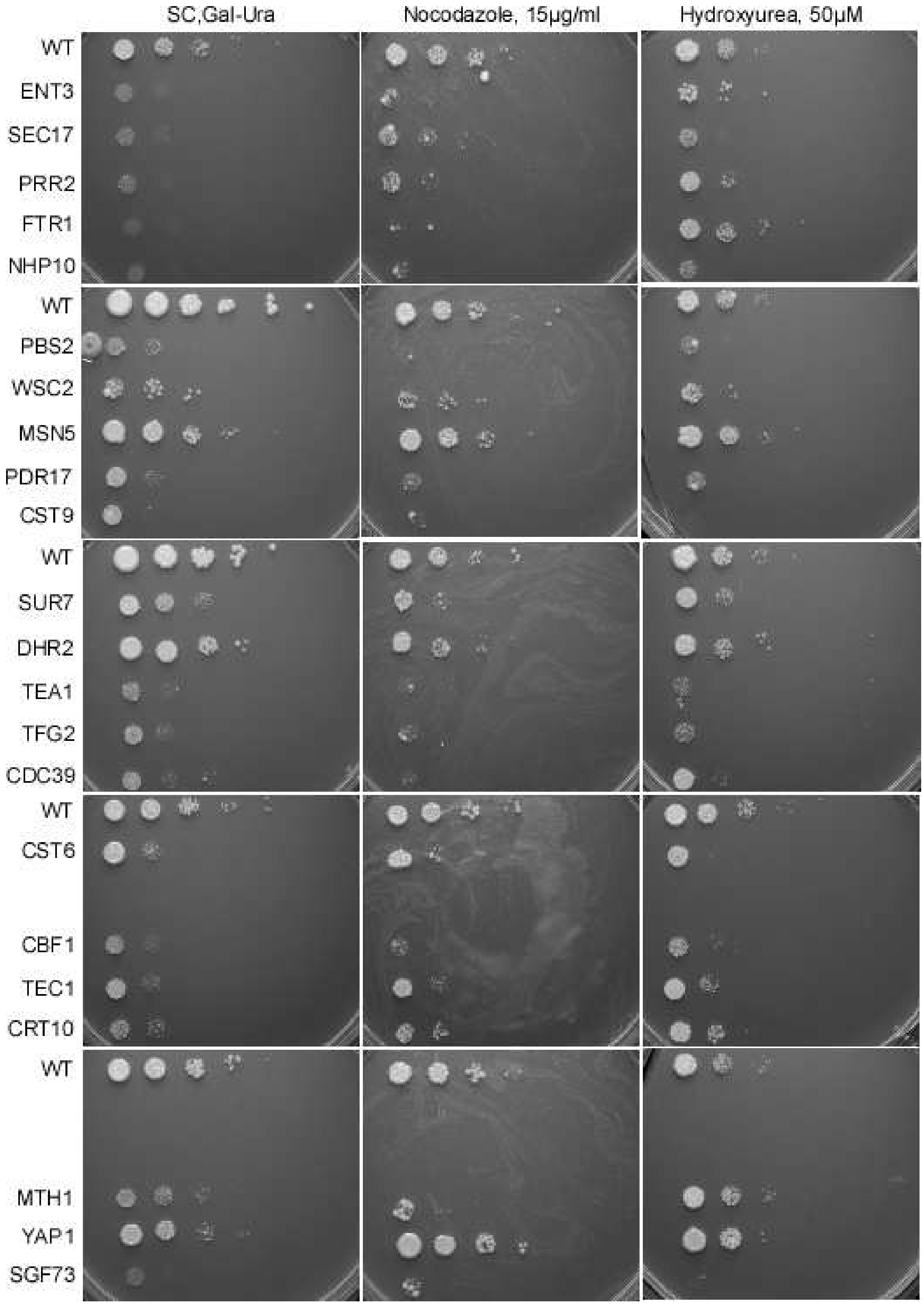
SC,Gal-Ura
Nocodazole, 15µg/ml
Hydroxyurea, 50µM
WT
ENT3
SEC17
PRR2
FTR1
NHP10
WT
PBS2
WSC2
MSN5
PDR17
CST9
WT
SUR7
DHR2
TEA1
TFG2
CDC39
WT
CST6
CBF1
TEC1
CRT10
WT
MTH1
YAP1
SGF73

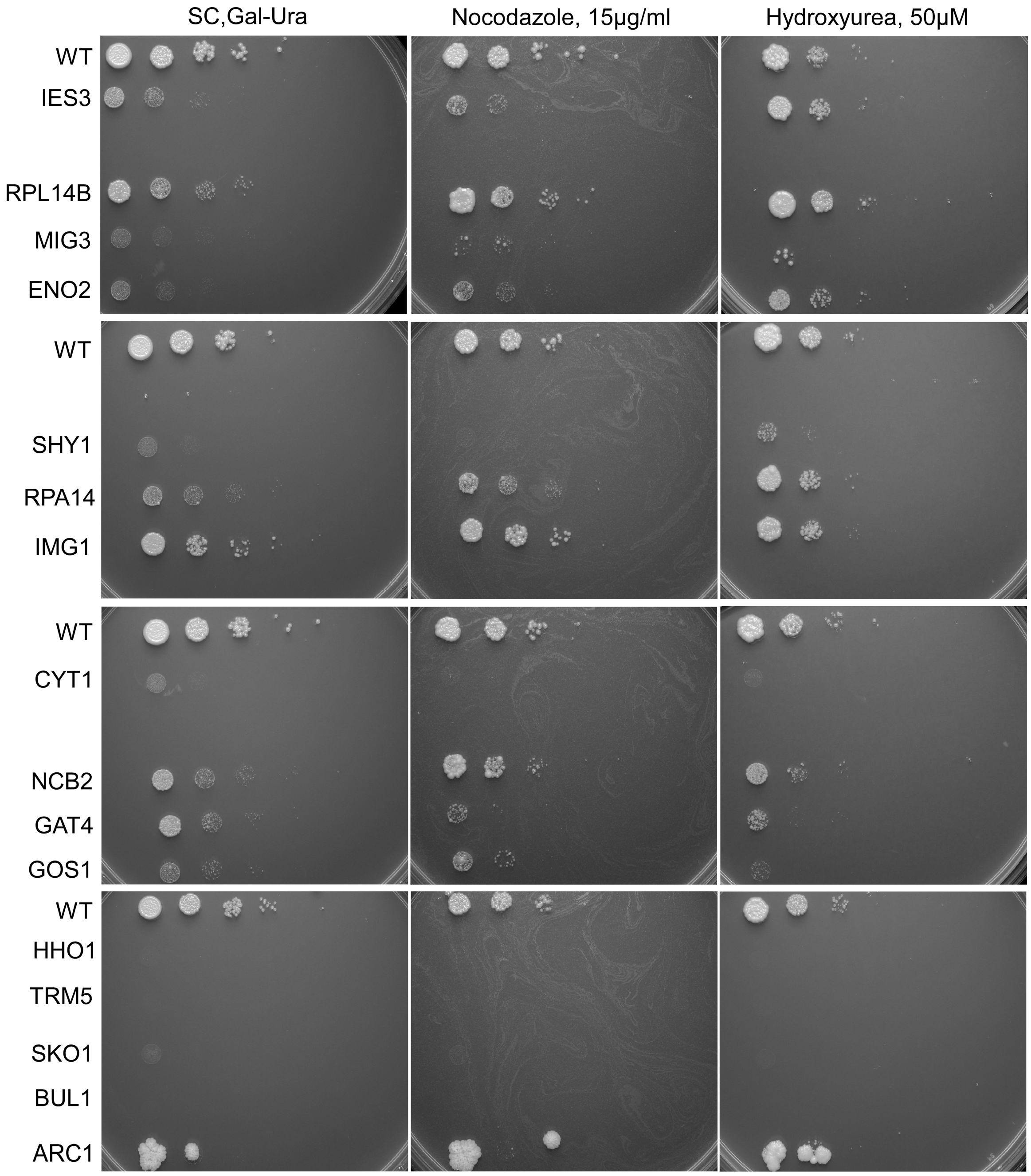
SC,Gal-Ura
Nocodazole, 15µg/ml
Hydroxyurea, 50µM
WT
IES3
RPL14B
MIG3
ENO2
WT
SHY1
RPA14
IMG1
WT
CYT1
NCB2
GAT4
GOS1
WT
HHO1
TRM5
SKO1
BUL1
ARC1
