# Supplementary material for: Mechanisms of Cell Cycle Control Revealed by a Systematic and Quantitative Overexpression Screen in S. cerevisiae
Source: PLoS Genet. 2008 Jul 11;4(7):e1000120. doi: 10.1371/journal.pgen.1000120 (PMC2438615; doi:10.1371/journal.pgen.1000120)
Supplement: Figure S3 — Protein expression is significantly induced in overexpression strains, even for proteins expressed natively at high levels. (0.14 MB PDF) [file pgen.1000120.s003.pdf]

Supplemental Figure 3: Protein expression is significantly induced in overexpression strains, even for proteins expressed natively at high levels. To verify overexpression, we performed Western blot analysis of 5 proteins spanning native steady-state expression levels from 172 to 871,000 copies/cell [1], measuring their native expression levels and their levels when expressed on the 2 micron plasmid under control of the inducible GAL1 promoter. To compare native and GAL1-controlled expression levels, we employed two strains for each protein, the GAL1 overexpression strain employed in this study [2], and a strain expressing a TAP-tagged version of the protein (which includes the protein A domain) under control of the native promoter and in the proper genomic locus [1]. Strains were cultured as described in Methods except that the medium for TAP strains was supplemented with 20ug/ml uracil. Equal numbers of cells (total 2 OD_{600}) for each strain were lysed in 50ul sample buffer. 5ul sample was loaded onto SDS-PAGE gel for detection of Urn1 and Rfa1 proteins; 10ul sample was loaded for detection of San1 protein. For high native abundance proteins, protein samples were diluted 10-fold (Arc1) or 100-fold (Yef3) and 5ul sample was loaded onto an SDS-PAGE gel. As both the ORF overexpression plasmid-encoded protein and the TAP-tagged protein contain protein A domains, both could be detected by the PAP antibody (Rockland Immunochemicals, Inc.) and luminol (Santa Cruz Biotechnology, Inc.). Protein abundance was quantified with ImageJ software. The ratio of protein levels in each TAP strain to the protein levels in the corresponding ORF strain was calculated and is shown on the top of each bar.

References

1. Ghaemmaghami S, Huh WK, Bower K, Howson RW, Belle A, et al. (2003) Global analysis of protein expression in yeast. Nature 425: 737-741.
2. Gelperin DM, White MA, Wilkinson ML, Kon Y, Kung LA, et al. (2005) Biochemical and genetic analysis of the yeast proteome with a movable ORF collection. Genes Dev 19: 2816-2826.

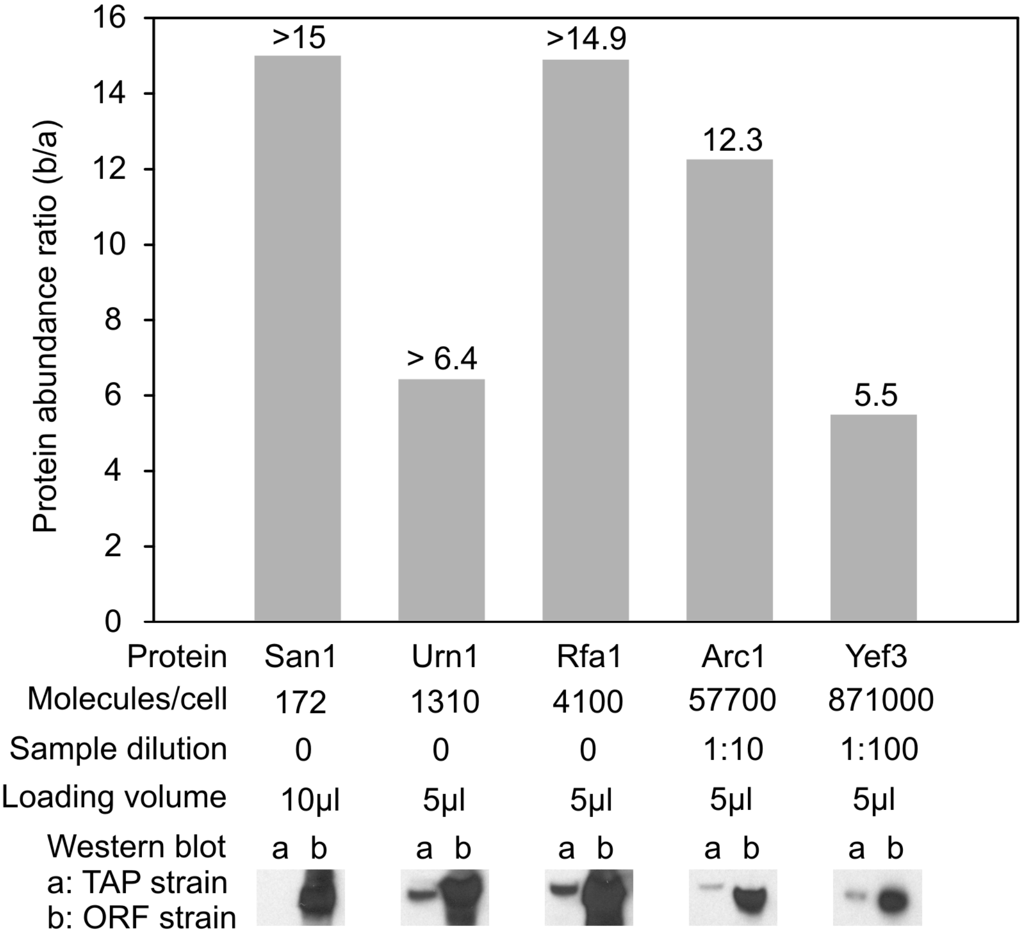

Protein abundance ratio (b/a)
16
14
12
10
8
6
4
2
0
>15
> 6.4
>14.9
12.3
5.5
Protein
San1
Urn1
Rfa1
Arc1
Yef3
Molecules/cell
172
1310
4100
57700
871000
Sample dilution
0
0
0
1:10
1:100
Loading volume
10µl
5µl
5µl
5µl
5µl
Western blot
a b
a b
a b
a b
a b
a: TAP strain
b: ORF strain
