# Supplementary material for: Mechanisms of Cell Cycle Control Revealed by a Systematic and Quantitative Overexpression Screen in S. cerevisiae
Source: PLoS Genet. 2008 Jul 11;4(7):e1000120. doi: 10.1371/journal.pgen.1000120 (PMC2438615; doi:10.1371/journal.pgen.1000120)
Supplement: Figure S4 — Overlap of identified genes with previous large-scale studies. (0.15 MB PDF) [file pgen.1000120.s004.pdf]

Supplemental Figure 4: Overlap of identified genes with previous large-scale studies. A Venn diagram illustrates the intersection of genes identified in this study as delaying cell cycle progression when overexpressed with those genes identified by Sopko *et al.* [1] and Stevenson *et al.* [2]. The intersection is statistically significant ($p < 0.05$, hypergeometric probability), but small, likely due to differences in the screens themselves (e.g., selecting genes for analysis based on overexpressing-induced growth defects [1,2] versus comprehensive flow cytometry), in the overexpression strains used, and in the presence of epitope tags (e.g., such as by addition of protein A tags to the proteins analyzed in this work).

References

1. Sopko R, Huang D, Preston N, Chua G, Papp B, et al. (2006) Mapping pathways and phenotypes by systematic gene overexpression. Mol Cell 21: 319-330.
2. Stevenson LF, Kennedy BK, Harlow E (2001) A large-scale overexpression screen in Saccharomyces cerevisiae identifies previously uncharacterized cell cycle genes. Proc Natl Acad Sci U S A 98: 3946-3951.

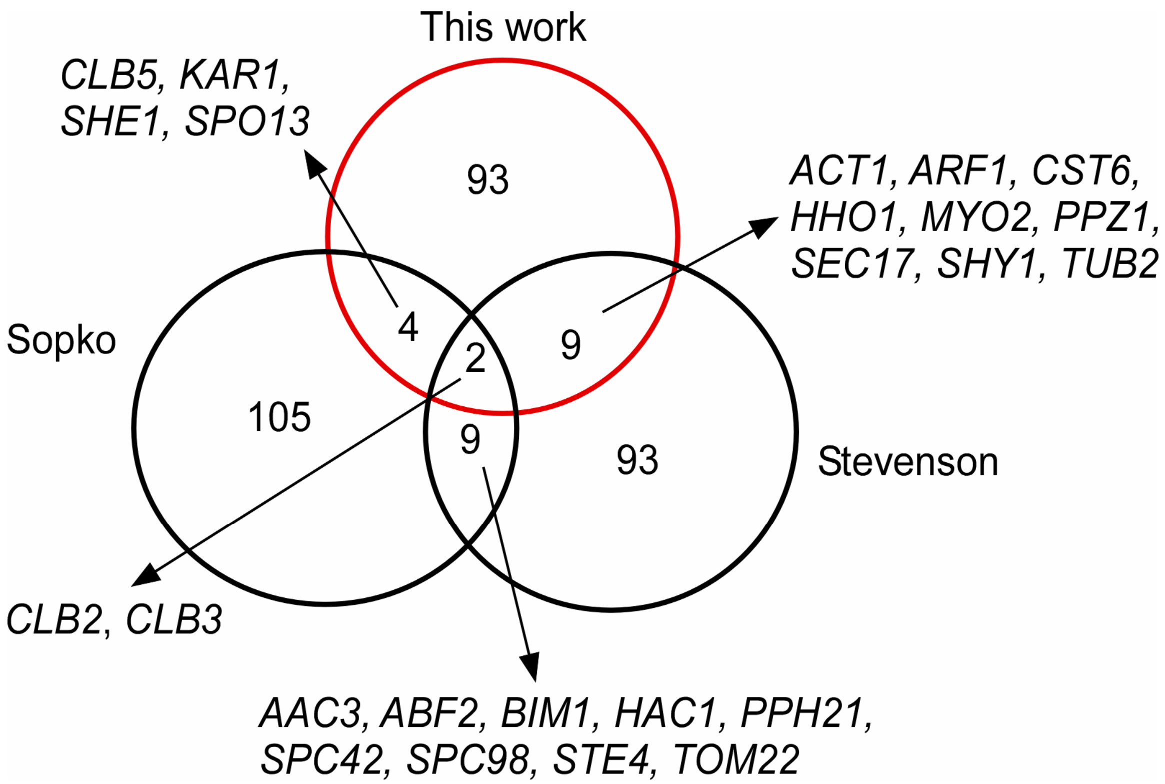
This work
CLB5, KAR1,
SHE1, SPO13
93
ACT1, ARF1, CST6,
HHO1, MYO2, PPZ1,
SEC17, SHY1, TUB2
Sopko
4
2
9
105
9
93
Stevenson
CLB2, CLB3
AAC3, ABF2, BIM1, HAC1, PPH21,
SPC42, SPC98, STE4, TOM22
