# Supplementary material for: Mechanisms of Cell Cycle Control Revealed by a Systematic and Quantitative Overexpression Screen in S. cerevisiae
Source: PLoS Genet. 2008 Jul 11;4(7):e1000120. doi: 10.1371/journal.pgen.1000120 (PMC2438615; doi:10.1371/journal.pgen.1000120)
Supplement: Table S1 — 108 yeast ORFs causing cell cycle defects when overexpressed. (0.03 MB PDF) [file pgen.1000120.s005.pdf]

Supplemental Table 1: Yeast ORFs causing cell cycle defects when overexpressed

21 ORF strains in G1 category listed with corresponding measurements of bud size distributions. Numbers indicate percentages of cells with no bud, small bud, and large bud, based upon *N* ~ 100 cells / strain. Bold: genes newly implicated in cell cycle progression; * : dubious ORFs.

Systematic name	Standard name	% unbudded cells	% small budded cells	% large budded cells
**YLR052W**	**IES3**	0.78	0.01	0.21
**YOR131C**	**YOR131C**	0.81	0.06	0.13
**YHL001W**	**RPL14B**	0.74	0.15	0.11
**YER028C**	**MIG3**	0.67	0.14	0.2
**YHR174W**	**ENO2**	0.74	0.13	0.13
**YDR117C**	**TMA64**	0.77	0.08	0.15
YGR112W	SHY1	0.74	0.06	0.23
**YDR156W**	**RPA14**	0.76	0.01	0.23
**YCR046C**	**IMG1**	0.72	0.08	0.21
**YDR493W**	**YDR493W**	0.79	0	0.21
**YOR065W**	**CYT1**	0.79	0	0.21
**YPR152C**	**YPR152C**	0.72	0.12	0.16
**YDR397C**	**NCB2**	0.8	0.07	0.13
**YIR013C**	**GAT4**	0.69	0.03	0.28
**YHL031C**	**GOS1**	0.77	0.02	0.21
**YPL127C**	**HHO1**	Not included	Not included	Not included
YHR070W	TRM5	0.92	0.06	0.02
**YNL167C**	**SKO1**	0.91	0.05	0.05
**YMR275C**	**BUL1**	0.97	0	0.03
**YGL105W**	**ARC1**	0.81	0.08	0.11
***YLL066W-B**	***YLL066W-B**	0.73	0.09	0.17
Empty vector	Empty vector	0.57	0.15	0.28

87 ORF strains in G2 category are listed with the measurements of bud size and nuclear morphology. Numbers represent percentages of cells with no bud, small bud, and large bud, based on *N* ~ 100 cells / strain, as well as percentages of large-budded cells in three categories of nuclear morphology, based on *N* ~ 50 large-budded cells / strain. The three categories of nuclear morphology are:

Class I: an undivided nucleus in one cell body of large budded cell

Class II: an undivided nucleus in bud neck of large budded cell

Class III: divided nuclei separated into two cell bodies

Bold: genes newly implicated in cell cycle progression; * : dubious ORFs.

Systematic name	Standard name	% unbudded cells	% small budded cells	% large budded cells	% class I	% class II	% class III
YKL052C	ASK1	0.67	0.11	0.21	0.11	0.14	0.75
***YBR131C-A**	***YBR131C-A**	0.53	0.16	0.31	0.24	0.22	0.53
YOR257W	CDC31	0.59	0.13	0.28	0.18	0.13	0.69
YCR093W	CDC39	0.33	0.12	0.55	0.12	0.17	0.71
**YGR206W**	**YGR206W**	0.57	0.07	0.37	0.29	0.09	0.63
**YML055W**	**SPC2**	0.53	0.05	0.42	0.1	0.07	0.83
YHR172W	SPC97	0.33	0.05	0.62	0.14	0.77	0.09
**YIL138C**	**TPM2**	0.52	0.05	0.43	0.04	0.18	0.78
YBL050W	SEC17	0.52	0.11	0.37	0.13	0.26	0.61
YOR326W	MYO2	0.57	0.12	0.31	0.15	0.22	0.63
**YNL264C**	**PDR17**	0.38	0.11	0.51	0.13	0.27	0.6
**YDR277C**	**MTH1**	0.41	0.09	0.5	0.04	0.38	0.58
***YLR123C**	***YLR123C**	0.6	0.1	0.3	0.13	0.2	0.67
**YML052W**	**SUR7**	0.39	0.18	0.43	0.17	0.26	0.57
YHR014W	SPO13	0.53	0.05	0.42	0.17	0.27	0.56
**YHR002W**	**LEU5**	0.52	0.06	0.42	0.07	0.24	0.69
**YLR394W**	**CST9**	0.52	0.09	0.39	0.18	0.28	0.54
**YJR060W**	**CBF1**	0.44	0.05	0.51	0.13	0.54	0.33
**YCL026C-A**	**FRM2**	0.51	0.09	0.4	0.17	0.4	0.43
**YPR015C**	**YPR015C**	0.41	0.11	0.48	0.03	0.68	0.29
**YOR286W**	**FMP31**	0.5	0.14	0.36	0.03	0.09	0.88
YGR091W	PRP31	0.34	0.16	0.49	0.12	0.16	0.72
**YDL002C**	**NHP10**	0.51	0.09	0.4	0.03	0.13	0.83
***YJL077W-A**	***YJL077W-A**	0.55	0.06	0.39	0.03	0.21	0.76
**YML007W**	**YAP1**	0.35	0.17	0.48	0.11	0.36	0.53
**YER145C**	**FTR1**	0.48	0.13	0.39	0.05	0.09	0.86
**YLR149C**	**YLR149C**	0.61	0.1	0.29	0.04	0.25	0.71
**YJL012C**	**VTC4**	0.5	0.06	0.44	0.09	0.11	0.8
**YLR341W**	**SPO77**	0.49	0.07	0.44	0.07	0.27	0.66
YNL188W	KAR1	0.54	0.02	0.43	0.1	0.12	0.79
YOR195W	SLK19	0.45	0.08	0.47	0.03	0.39	0.58
YGR109C	CLB6	0.44	0.17	0.39	0.08	0.35	0.58
YBR211C	AME1	0.49	0.1	0.41	0.05	0.3	0.65
**YDR245W**	**MNN10**	0.44	0.11	0.45	0	0.16	0.84
**YDR033W**	**MRH1**	0.4	0.18	0.42	0.03	0.13	0.84

YJL030W	MAD2	0.54	0.1	0.36	0.02	0.14	0.84
**YDR091C**	**RLI1**	0.41	0.06	0.53	0.04	0.34	0.62
**YIR001C**	**SGN1**	0.32	0.08	0.6	0.06	0.29	0.64
**YKL078W**	**DHR2**	0.45	0.11	0.43	0.02	0.22	0.76
**YPR190C**	**RPC82**	0.51	0.14	0.35	0.02	0.59	0.39
**YDR266C**	**YDR266C**	0.45	0.07	0.48	0.04	0.25	0.71
**YDL214C**	**PRR2**	0.5	0.09	0.42	0.08	0.15	0.78
**YDR001C**	**NTH1**	0.44	0.1	0.45	0.05	0.61	0.33
**YIR016W**	**YIR016W**	0.41	0.08	0.51	0	0.23	0.77
**YBR083W**	**TEC1**	0.29	0.22	0.49	0.02	0.19	0.79
**YKR067W**	**GPT2**	0.54	0.15	0.31	0.07	0.18	0.75
**YHR131C**	**YHR131C**	0.53	0.08	0.39	0.17	0.13	0.71
**YFL022C**	**FRS2**	0.46	0.1	0.44	0.07	0.23	0.7
**YDR143C**	**SAN1**	0.4	0.1	0.5	0.12	0.18	0.71
YPL174C	NIP100	0.26	0.05	0.69	0.25	0.59	0.16
**YOR002W**	**ALG6**	0.45	0.11	0.44	0.11	0.21	0.68
**YNL283C**	**WSC2**	0.35	0.17	0.49	0.13	0.13	0.73
**YPL247C**	**YPL247C**	0.39	0.11	0.49	0.16	0.27	0.58
**YJL031C**	**BET4**	0.45	0.18	0.37	0.11	0.36	0.52
YPR119W	CLB2	0.34	0.09	0.56	0.08	0.45	0.47
**YML053C**	**YML053C**	0.56	0.14	0.3	0.09	0.11	0.81
**YJL106W**	**IME2**	0.41	0.13	0.46	0.1	0.04	0.86
YMR199W	CLN1	Pseudohyphal growth					
**YML016C**	**PPZ1**	0.29	0.03	0.67	0.11	0.68	0.21
**YLR189C**	**ATG26**	0.38	0.11	0.51	0.04	0.14	0.82
YER007W	PAC2	0.39	0.12	0.49	0.2	0.42	0.38
**YEL022W**	**GEA2**	0.41	0.05	0.54	0.29	0.35	0.36
YDL192W	ARF1	0.36	0.12	0.52	0.05	0.12	0.83
**YDR335W**	**MSN5**	0.41	0.13	0.45	0.02	0.18	0.8
**YKR029C**	**SET3**	0.33	0.06	0.62	0.07	0.22	0.72
**YGR094W**	**VAS1**	0.49	0.12	0.39	0.07	0.17	0.76
YBL031W	SHE1	0.37	0.14	0.49	0.1	0.47	0.43
YGR005C	TFG2	0.51	0.07	0.42	0.12	0.18	0.71
**YGR109W-A**	**YGR109W-A**	0.36	0.17	0.48	0.11	0.18	0.71
**YPL116W**	**HOS3**	0.29	0.06	0.65	0.19	0.19	0.61
**YAR007C**	**RFA1**	0.13	0.04	0.82	0.2	0.73	0.06
**YDL093W**	**PMT5**	0.42	0.12	0.46	0.1	0.21	0.69
**YOL063C**	**CRT10**	0.47	0.11	0.42	0.11	0.17	0.71
**YJR125C**	**ENT3**	0.36	0.12	0.53	0.05	0.36	0.59
**YIL036W**	**CST6**	0.34	0.15	0.51	0.07	0.18	0.76
**YOR337W**	**TEA1**	0.26	0.08	0.66	0.12	0.46	0.42
**YOR007C**	**SGT2**	0.54	0.13	0.33	0	0.22	0.78
**YJL128C**	**PBS2**	0.42	0.13	0.44	0.09	0.18	0.73
**YIL158W**	**YIL158W**	0.36	0.1	0.54	0.16	0.25	0.59
**YHR177W**	**YHR177W**	0.42	0.16	0.43	0.02	0.23	0.74
**YGL066W**	**SGF73**	0.42	0.12	0.45	0.11	0.22	0.67

**YMR068W**	**AVO2**	0.43	0.09	0.49	0.13	0.55	0.32
**YER131W**	**RPS26B**	0.52	0.12	0.36	0.04	0.06	0.89
YFL037W	TUB2	0.24	0.04	0.72	0.47	0.34	0.19
YDL155W	CLB3	0.28	0.06	0.66	0.25	0.29	0.46
YPR120C	CLB5	0.3	0.09	0.62	0.21	0.31	0.49
YFL039C	ACT1	0.27	0.1	0.63	0.53	0.27	0.2
Empty vector	Empty vector	0.57	0.15	0.28	0.11	0.26	0.6
