# Supplementary material for: Mechanisms of Cell Cycle Control Revealed by a Systematic and Quantitative Overexpression Screen in S. cerevisiae
Source: PLoS Genet. 2008 Jul 11;4(7):e1000120. doi: 10.1371/journal.pgen.1000120 (PMC2438615; doi:10.1371/journal.pgen.1000120)
Supplement: Table S2 — Summary of 108 strains with cell cycle defects. (0.01 MB PDF) [file pgen.1000120.s006.pdf]

Supplemental Table 2: Summary of 108 strains with cell cycle defects.
* Genes in G1 category.

21 essential genes	26 known cdc genes	17 transcription factors	24 uncharacterized ORFs	48 others
*NCB2	*SHY1	*SKO1	*YLL066W-B	*HHO1
*TRM5	*TRM5	*GAT4	*YOR131C	*BUL1
ASK1	ASK1	*NCB2	*YPR152C	*GOS1
CDC31	CDC31	*MIG3	*TMA64	*RPL14B
CDC39	CDC39	TEA1	*YDR493W	*RPA14
SPC97	SPC97	TFG2	YGR206W	*ENO2
SEC17	SEC17	CDC39	YML053C	*ARC1
MYO2	MYO2	CST6	YPR015C	*CYT1
PRP31	SPO13	YPR015C	YHR131C	*IES3
KAR1	CLN1	CBF1	YLR123C	*IMG1
AME1	PRP31	TEC1	FRM2	RFA1
RLI1	KAR1	CRT10	YIL158W	HOS3
DHR2	SLK19	YDR266C	YHR177W	MRH1
RPC82	CLB6	YGR109W-A	YDR266C	SGN1
FRS2	AME1	MTH1	YGR109W-A	IME2
BET4	MAD2	YAP1	YIR016W	MNN10
VAS1	NIP100	SGF73	YLR149C	BET4
TFG2	CLB2		FMP31	NTH1
RFA1	CLB5		YPL247C	GEA2
TUB2	PAC2		YBR131C-A	GPT2
ACT1	ARF1		SHE1	RPS26B
	SHE1		YJL077W-A	SPC2
	TFG2		FMP31	SPO77
	CLB3		YDL159W-A	LEU5
	TUB2			TPM2
	ACT1			FRS2
	ASK1			RPC82
	CDC31			PMT5
				VTC4
				ALG6
				SGT2
				SET3
				VAS1
				SAN1
				AVO2
				ATG26
				ENT3
				SEC17
				PRR2
				FTR1
				NHP10
				PBS2
				WSC2

				MSN5
				PDR17
				CST9
				SUR7
				DHR2
