# Supplementary material for: Mechanisms of Cell Cycle Control Revealed by a Systematic and Quantitative Overexpression Screen in S. cerevisiae
Source: PLoS Genet. 2008 Jul 11;4(7):e1000120. doi: 10.1371/journal.pgen.1000120 (PMC2438615; doi:10.1371/journal.pgen.1000120)
Supplement: Table S3 — Comparison between over-expression and loss-of-function phenotypes. (0.01 MB PDF) [file pgen.1000120.s007.pdf]

Supplemental Table 3: Comparison between over-expression and loss-of-function phenotypes

Genes in G1 category

Overexpression only	Overexpression ∩ SCMD	Genes not included in SCMD
BUL1	YDR493W	TRM5
SKO1		NCB2
YOR131C		
ARC1		
CYT1		
IES3		
TMA64		
GOS1		
RPA14		

Genes in G2 category

Overexpression only	Overexpression ∩ SCMD	Genes not included in SCMD
NIP100	PDR17	TUB2
PPZ1	MNN10	ACT1
TEA1	ARF1	SPC97
CLB3		CDC39
HOS3		GEA2
SET3		RLI1
CLB5		PRP31
SGN1		YGR109W-A
CLB2		VTC4
YIL158W		RFA1
ENT3		
YIR016W		
CST6		
CBF1		
ATG26		
SAN1		
MTH1		
TEC1		
YPL247C		
SHE1		
PAC2		
AVO2		
WSC2		
YAP1		
YPR015C		
YDR266C		
SLK19		

IME2		
PMT5		
MSN5		
NTH1		
SGF73		
ALG6		
PBS2		
