# Supplementary material for: Mechanisms of Cell Cycle Control Revealed by a Systematic and Quantitative Overexpression Screen in S. cerevisiae
Source: PLoS Genet. 2008 Jul 11;4(7):e1000120. doi: 10.1371/journal.pgen.1000120 (PMC2438615; doi:10.1371/journal.pgen.1000120)
Supplement: Table S4 — Genes whose overexpression induces slow growth, drug sensitivity. (0.01 MB PDF) [file pgen.1000120.s008.pdf]

Supplemental Table 4: Genes whose overexpression induces slow growth, drug sensitivity. 77 new genes leading to cell cycle defects upon overexpression and 3 positive controls were tested for slow growth phenotypes and sensitivity to nocodazole (NOC: 15µg/ml) and hydroxyurea (HU: 50µM).

Genes in the G1 category:

SC_GAL	No slow growth on SC_GAL	SC_GAL_NOC	SC_GAL_HU	NOC and HU
IES3	IMG1	YDR493W	YOR131C	MIG3
YOR131C			YPR152C	GAT4
RPL14B			GOS1	
MIG3				
ENO2				
TMA64				
RPA14				
YDR493W				
CYT1				
YPR152C				
NCB2				
GAT4				
GOS1				
HHO1				
SKO1				
BUL1				
ARC1				

Gene in the G2/M category:

SC_GAL	No slow growth on SC_GAL	SC_GAL_NOC	SC_GAL_HU	Resistant to HU	NOC and HU
YGR206W	DHR2	YML053C	LEU5	FRM2	YHR131C
SPC2	GPT2	YPR015C	WSC2	YIR016W	YPL247C
TPM2	YGR109W-A	YLR149C	SGF73	PMT5	RFA1
PDR17		MRH1		FTR1	MNN10
MTH1		HOS3			BET4
SUR7		SGN1			VTC4
LEU5		IME2			SET3
CST9		YDR266C			SGT2
CBF1		GEA2			ALG6
FRM2		SPO77			CST9
YPR015C		RLI1			SUR7
FMP31		VAS1			
NHP10		AVO2			
YAP1		ATG26			
FTR1		FMP31			
YLR149C		FTR1			

VTC4		PBS2			
SPO77		PDR17			
MNN10		TEA1			
MRH1		CBF1			
RLI1		MTH1			
SGN1					
RPC82					
YDR266C					
PRR2					
NTH1					
YIR016W					
TEC1					
YHR131C					
FRS2					
SAN1					
ALG6					
WSC2					
YPL247C					
BET4					
YML053C					
IME2					
ATG26					
GEA2					
MSN5					
SET3					
VAS1					
HOS3					
RFA1					
PMT5					
CRT10					
ENT3					
CST6					
TEA1					
SGT2					
PBS2					
YIL158W					
YHR177W					
SGF73					
AVO2					
RPS26B					
