# Supplementary material for: Mechanisms of Cell Cycle Control Revealed by a Systematic and Quantitative Overexpression Screen in S. cerevisiae
Source: PLoS Genet. 2008 Jul 11;4(7):e1000120. doi: 10.1371/journal.pgen.1000120 (PMC2438615; doi:10.1371/journal.pgen.1000120)
Supplement: Table S5 — Genes upregulated following overexpression of SKO1. (0.02 MB PDF) [file pgen.1000120.s009.pdf]

Supplemental Table 5: Genes upregulated following overexpression of *SKO1*.

Bold: p-value <0.01. Red bold: p-value <0.01 and involved in the pheromone response pathway.

Systematic name	Standard name	p-value
**YIL082W**	**YIL082W**	**1.13E-10**
**YGR109W-A**	**YGR109W-A**	**1.58E-10**
**YILWTY3-1A**	**YILWTY3-1A**	**4.12E-10**
**YIR034C**	**LYS1**	**1.42E-09**
**YILWTY3-1C**	**YILWTY3-1C**	**3.33E-09**
**YHL016C**	**DUR3**	**6.00E-09**
**YCL055W**	**KAR4**	**1.14E-08**
**YGL032C**	**AGA2**	**1.62E-08**
**YNR044W**	**AGA1**	**1.78E-08**
**YDR461W**	**MFA1**	**7.70E-08**
**YHR018C**	**ARG4**	**1.68E-07**
**YHLCSIGMA1**	**YHLCSIGMA1**	**3.49E-07**
**YFL026W**	**STE2**	**4.45E-07**
**YCL018W**	**LEU2**	**1.48E-06**
**YILWTY3-1B**	**YILWTY3-1B**	**1.57E-06**
**YKR093W**	**PTR2**	**2.40E-06**
**YOL058W**	**ARG1**	**2.96E-06**
**YBLCSIGMA1**	**YBLCSIGMA1**	**3.21E-06**
**YOR225W**	**YOR225W**	**5.63E-06**
**YKL035W**	**UGP1**	**1.15E-05**
**YOR226C**	**ISU2**	**1.21E-05**
**YIL015W**	**BAR1**	**1.23E-05**
**YBR256C**	**RIB5**	**1.28E-05**
**YDR171W**	**HSP42**	**1.32E-05**
**YGL184C**	**STR3**	**2.55E-05**
**YIL082W-A**	**YIL082W-A**	**4.58E-05**
**YJR152W**	**DAL5**	**7.45E-05**
**YBR104W**	**YMC2**	**8.03E-05**
**YDR309C**	**GIC2**	**9.58E-05**
**YPL092W**	**SSU1**	**0.000106**
**YMR058W**	**FET3**	**0.000115**
**YCL027W**	**FUS1**	**0.000135**
**YEL065W**	**SIT1**	**0.000139**
**YMR136W**	**GAT2**	**0.000142**
**YIL114C**	**POR2**	**0.000231**

**YER069W**	**ARG5,6**	**0.000267**
**YILWTY3-1D**	**YILWTY3-1D**	**0.000276**
**YDR158W**	**HOM2**	**0.000287**
**YIL117C**	**PRM5**	**0.000294**
**YBR043C**	**QDR3**	**0.00031**
**YCL030C**	**HIS4**	**0.000335**
**YPL156C**	**PRM4**	**0.000385**
**YOR383C**	**FIT3**	**0.000457**
**YGL053W**	**PRM8**	**0.000488**
**YPL250C**	**ICY2**	**0.000543**
**YKR039W**	**GAP1**	**0.000623**
**YGR065C**	**VHT1**	**0.000664**
**YJR153W**	**PGU1**	**0.000806**
**YBR169C**	**SSE2**	**0.000891**
**YKR069W**	**MET1**	**0.001082**
**YDR043C**	**NRG1**	**0.001268**
**YGR019W**	**UGA1**	**0.001366**
**YGL009C**	**LEU1**	**0.001488**
**YPL265W**	**DIP5**	**0.001536**
**YIR032C**	**DAL3**	**0.001597**
**YBR092C**	**PHO3**	**0.001618**
**YDL170W**	**UGA3**	**0.001818**
**YKL120W**	**OAC1**	**0.001843**
**YHR208W**	**BAT1**	**0.003597**
**YBR040W**	**FIG1**	**0.003645**
**YOR203W**	**YOR203W**	**0.003689**
**YBR156C**	**SLI15**	**0.004268**
**YHR180W**	**YHR180W**	**0.004604**
**YGL096W**	**TOS8**	**0.004978**
**YLL026W**	**HSP104**	**0.005045**
**YKL062W**	**MSN4**	**0.005224**
**YJR150C**	**DAN1**	**0.005236**
**YDL198C**	**GGC1**	**0.005669**
**YFL047W**	**RGD2**	**0.005681**
**YIL108W**	**YIL108W**	**0.00576**
**YCR089W**	**FIG2**	**0.006272**
**YDR533C**	**HSP31**	**0.006641**
YOR202W	HIS3	0.011976
YMR096W	SNZ1	0.016113
YGR142W	BTN2	0.018001
YFR030W	MET10	0.021845
YKL178C	STE3	0.029495
YPL276W	YPL276W	0.029906
YGR109W-B	YGR109W-B	0.031031

YLR237W	THI7	0.034019
YJL170C	ASG7	0.038652
YNL167C	SKO1	0.04157
YKL211C	TRP3	0.047914
YHR126C	YHR126C	0.054046
YPL278C	YPL278C	0.069839
YKR034W	DAL80	0.070488
YBL086C	YBL086C	0.102797
YIL080W	YIL080W	0.131767
YHL036W	MUP3	0.141744
YOL119C	MCH4	0.144385
YOR193W	PEX27	0.169166
YNL065W	AQR1	0.183735
YBR214W	SDS24	0.188756
YIL120W	QDR1	0.188829
YNL142W	MEP2	0.207984
YDL085W	NDE2	0.219352
YIL164C	NIT1	0.244444
YLR267W	BOP2	0.326351
YNL042W	BOP3	0.328169
YGR055W	MUP1	0.329911
YML116W	ATR1	0.374456
YML128C	MSC1	0.406651
YLR307W	CDA1	0.429131
YDR442W	YDR442W	0.433514
YKR105C	YKR105C	0.444108
YMR182C	RGM1	0.45413
YGL164C	YRB30	0.455562
YIL160C	POT1	0.464897
YNL279W	PRM1	0.468232
YMR120C	ADE17	0.471552
YFL053W	DAK2	0.498348
YLR266C	PDR8	0.505084
YML047C	PRM6	0.506647
YMR232W	FUS2	0.523272
YLR178C	TFS1	0.528707
YJR004C	SAG1	0.540445
YPL280W	HSP32	0.554166
YGLCDELTA5	YGLCDELTA5	0.569083
YMR322C	SNO4	0.610928
YPR136C	#N/A	0.624643
YMR095C	SNO1	0.627073
YFL055W	AGP3	0.630934
YGL181W	GTS1	0.631746

YNR067C	DSE4	0.631772
YIL113W	SDP1	0.649168
YBR147W	YBR147W	0.653764
YER185W	YER185W	0.664736
YOL158C	ENB1	0.664814
YML048W-A	YML048W-A	0.707211
YPL187W	MF(ALPHA)1	0.712439
YOR011W	AUS1	0.737899
YHL044W	YHL044W	0.770039
YMR057C	YMR057C	0.789247
YPR160W	GPH1	0.792923
YIR017C	MET28	0.838734
YIR013C	GAT4	0.841098
YBL075C	SSA3	0.858576
YHR084W	STE12	0.872093
YNL229C	URE2	0.872634
YMR173W-A	YMR173W-A	0.888746
YKL177W	YKL177W	0.916374
YDL245C	HXT15	0.922828
YLR452C	SST2	0.957528
YOR389W	YOR389W	0.962837
YIL037C	PRM2	0.982168
RDN18-1A	RDN18-1A	0.990424
YJR154W	YJR154W	1.006351
YPR159W	KRE6	1.026654
YOR106W	VAM3	1.054805
YHR168W	MTG2	1.066644
YMR198W	CIK1	1.081809
YFR023W	PES4	1.089436
YNL165W	YNL165W	1.102544
YNR069C	BSC5	1.114832
YOL055C	THI20	1.120593
YNR064C	YNR064C	1.126487
YMR065W	KAR5	1.137217
YLL028W	TPO1	1.140447
YJL203W	PRP21	1.141816
YDR273W	DON1	1.16255
REP2	REP2	1.163069
YNL123W	NMA111	1.164211
YPR153W	YPR153W	1.167228
RDN5-1	RDN5-1	1.219257
YGL033W	HOP2	1.224666
YER060W-A	FCY22	1.230945
YJL108C	PRM10	1.249204

YOR329C	SCD5	1.26001
YHRCDELTA4	YHRCDELTA4	1.263241
YPL017C	IRC15	1.327718
YLR218C	YLR218C	1.32931
SNR70	SNR70	1.338083
YGL214W	YGL214W	1.371733
YNL231C	PDR16	1.435346
YMR089C	YTA12	1.439261
YNL127W	FAR11	1.454467
YER188W	YER188W	1.564968
