# Supplementary material for: Mechanisms of Cell Cycle Control Revealed by a Systematic and Quantitative Overexpression Screen in S. cerevisiae
Source: PLoS Genet. 2008 Jul 11;4(7):e1000120. doi: 10.1371/journal.pgen.1000120 (PMC2438615; doi:10.1371/journal.pgen.1000120)
Supplement: Table S6 — Over-expression strains appearing diploid or 3C. (0.04 MB PDF) [file pgen.1000120.s010.pdf]

Supplemental Table 6: Over-expression strains appearing diploid or 3C. These strains failed to show Gal-dependence on the phenotype and were omitted from further analysis.

Diploid category		3C category	
**Systematic name**	**Standard name**	**Systematic name**	**Standard name**
YGR174C	CBP4	YGR120C	COG2
YKL176C	LST4	YEL017C-A	PMP2
YNL061W	NOP2	YBR179C	FZO1
YBR103W	SIF2	YKR044W	UIP5
YAR003W	SWD1	YNL131W	TOM22
YGR013W	SNU71	YLR126C	YLR126C
YHR149C	SKG6	YJL050W	MTR4
YOR348C	PUT4	YMR313C	TGL3
YKL047W	YKL047W	YCL063W	VAC17
YLR336C	SGD1	YKL160W	ELF1
YLL003W	SFI1	YNL114C	YNL114C
YKL163W	PIR3	YNR074C	AIF1
YEL060C	PRB1	YOL072W	THP1
YNL097C	PHO23	YLR139C	SLS1
YFR037C	RSC8	YBR239C	YBR239C
YGL023C	PIB2	YHR169W	DBP8
YGR051C	YGR051C	YLR062C	YLR062C
Q0250	COX2	YGL062W	PYC1
YBL026W	LSM2	YPR051W	MAK3
YMR117C	SPC24	YJR147W	HMS2
YLR148W	PEP3	YOR161C-A	YOR161C-A
YML047C	PRM6	YLR275W	SMD2
YML047C	PRM6	YER074W-A	YOS1
YJR017C	ESS1	YPL002C	SNF8
YPL161C	BEM4	YER186C	YER186C
YGR122C-A	YGR122C-A	YDR339C	FCF1
YLR365W	YLR365W	YNL109W	YNL109W
YKR060W	UTP30	YBR258C	SHG1
YGR106C	YGR106C	YGL242C	YGL242C
YJR097W	JJJ3	YNL190W	YNL190W
YDR540C	YDR540C	YJR107W	YJR107W
YLR068W	FYV7	YKL162C	YKL162C
YMR267W	PPA2	YHR098C	SFB3

YBR087W	RFC5	YNL059C	ARP5
YDR363W-A	SEM1	YEL045C	YEL045C
YPL152W	RRD2	YDR006C	SOK1
YER037W	PHM8	YJL194W	CDC6
YDL114W-A	YDL114W-A	YLL050C	COF1
YDR149C	YDR149C	YMR302C	PRP12
YER185W	YER185W	YPL248C	GAL4
YGL101W	YGL101W	YCR067C	SED4
YBR283C	SSH1	YEL052W	AFG1
YMR270C	RRN9	YMR243C	ZRC1
YCR009C	RVS161	YML054C	CYB2
YNL180C	RHO5	YPR193C	HPA2
YGR104C	SRB5	YMR149W	SWP1
YLR421C	RPN13	YBR234C	ARC40
YGR213C	RTA1	YPL206C	YPL206C
YPR009W	SUT2	YNL134C	YNL134C
YNL213C	YNL213C	YMR193W	MRPL24
YOL048C	YOL048C		
YOR295W	UAF30		
YML001W	YPT7		
YNL159C	ASI2		
YAR030C	YAR030C		
YPL045W	VPS16		
YER098W	UBP9		
YDR058C	TGL2		
YBR143C	SUP45		
YDR222W	YDR222W		
YKR014C	YPT52		
YGR267C	FOL2		
YDR045C	RPC11		
YLR424W	NTR1		
YKL135C	APL2		
YDL100C	GET3		
YFL030C-A	YFL030C-A		
YEL001C	YEL001C		
YGL052W	YGL052W		
YDR454C	GUK1		
YGL090W	LIF1		
YPL244C	HUT1		
YCR044C	PER1		
YLR441C	RPS1A		
YIL118W	RHO3		
YNL097C-B	YNL097C-B		

YDL243C	AAD4		
YDR177W	UBC1		
YNL056W	YNL056W		
YOR189W	IES4		
YNL099C	OCA1		
YPL044C	YPL044C		
YPL103C	FMP30		
YNL165W	YNL165W		
YHR058C	MED6		
YMR286W	MRPL33		
YNL030W	HHF2		
YNL215W	IES2		
YLR320W	MMS22		
YJR136C	YJR136C		
YML007C-A	YML007C-A		
YHL045W	YHL045W		
YJR071W	YJR071W		
YML071C	COG8		
YFL065C	YFL065C		
YMR274C	RCE1		
YDL071C	YDL071C		
YCR023C	YCR023C		
YIL173W	VTH1		
YMR214W	SCJ1		
YKR050W	TRK2		
YHR060W	VMA22		
YPL250C	ICY2		
YNR046W	TRM112		
YPR055W	SEC8		
YPR055W	SEC8		
YDR303C	RSC3		
YPR070W	MED1		
YPR070W	MED1		
YDL145C	COP1		
tORF6	tORF6		
YOR113W	AZF1		
YCL064C	CHA1		
YOL007C	YOL007C		
YGL038C	OCH1		
YBL089W	AVT5		
YPR106W	ISR1		
YHR198C	FMP22		
YLR301W	YLR301W		

YJL192C	SOP4		
YDR514C	YDR514C		
YGL104C	VPS73		
YGL014W	PUF4		
YNL040W	YNL040W		
YEL012W	UBC8		
YLR005W	SSL1		
YGR251W	YGR251W		
YLR400W	YLR400W		
YMR090W	YMR090W		
YNL158W	PGA1		
YOR314W	YOR314W		
YMR120C	ADE17		
YDR441C	APT2		
YPR130C	YPR130C		
YPR084W	YPR084W		
YPR133C	SPN1		
YBR162W-A	YSY6		
YNL150W	YNL150W		
YPR099C	YPR099C		
YNL152W	YNL152W		
YOL032W	OPI10		
YOL032W	OPI10		
YPL041C	YPL041C		
YDR368W	YPR1		
YGL253W	HXK2		
YDR309C	GIC2		
YBR020W	GAL1		
YHR094C	HXT1		
YNL031C	HHT2		
YNL103W	MET4		
YJR069C	HAM1		
YBR248C	HIS7		
YOR142W	LSC1		
YER091C	MET6		
YBR015C	MNN2		
YGL077C	HNM1		
YNR072W	HXT17		
YPR159W	KRE6		
YLR332W	MID2		
YMR172W	HOT1		
YMR172W	HOT1		
YBL003C	HTA2		

YKL190W	CNB1		
YDL126C	CDC48		
YER029C	SMB1		
YER072W	VTC1		
YFL017W-A	SMX2		
YGR162W	TIF4631		
YBR206W	YBR206W		
YBL075C	SSA3		
YML021C	UNG1		
YER024W	YAT2		
YBR280C	YBR280C		
YER005W	YND1		
YOR324C	FRT1		
YLR413W	YLR413W		
YMR135C	GID8		
YOR356W	YOR356W		
YLR414C	YLR414C		
YNL077W	APJ1		
YLR190W	MMR1		
YLR077W	FMP25		
YDR281C	PHM6		
YJR063W	RPA12		
YKL059C	MPE1		
YHR021C	RPS27B		
YFR026C	YFR026C		
YHR122W	YHR122W		
YGL051W	MST27		
YJR014W	TMA22		
YKL169C	YKL169C		
YNL142W	MEP2		
YLR245C	CDD1		
YDR047W	HEM12		
YLR213C	CRR1		
YBR222C	PCS60		
YGL181W	GTS1		
YGR068C	YGR068C		
YER188W	YER188W		
YJL083W	TAX4		
YIL108W	YIL108W		
YDL033C	SLM3		
YOR223W	YOR223W		
YKL146W	AVT3		
YGL166W	CUP2		

YNL304W	YPT11		
YIL153W	RRD1		
YAR035W	YAT1		
YJL071W	ARG2		
YNL176C	YNL176C		
YBR298C-A	YBR298C-A		
YOR391C	HSP33		
YJL156W-A	YJL156W-A		
YCL031C	RRP7		
